# Age-Related Survival Disparities in Advanced Renal Carcinoma in the Immune Checkpoint Inhibitor Era using the SEER Database and Meta-analysis

**DOI:** 10.1038/s41598-025-06297-y

**Published:** 2025-12-03

**Authors:** Abdullah Al-Danakh, Yuli Jian, Linlin Yang, Mohammed Safi, Xinqing Zhu, Mohammed Alradhi, Bassam Askar, Salem Baldi, Qiwei Chen, Shujing Wang, Deyong Yang

**Affiliations:** 1 Department of Urology, First Affiliated Hospital of Dalian Medical University, Dalian, Liaoning China; 2 Department of Biochemistry and Molecular Biology, Institute of Glycobiology, Dalian Medical University, Dalian, China; 3 National Oncology Center, Sana’a, Yemen; 4 Department of Urology, Aljumhori Teaching Hospital Authority, Faculty of Medicine and Health Sciences, Sana’a University, Sana’a, Yemen; 5 Department of Urology, Al-shorta Hospital, Sana’a, Yemen; 6 Department of Clinical Laboratory Diagnostics, School of Medical Technology, Shaoyang University, Shaoyang, 422000 China; 7 Department of Surgery, Healinghands Clinic, Dalian, Liaoning China; 8 Department of Urology, Amran University, Amran, Yemen

**Keywords:** Advanced renal cell carcinoma, Immune checkpoint inhibitors, SEER, Meta-analysis, TNFSF15, Age-specific survival, Cancer, Cell biology, Computational biology and bioinformatics, Genetics, Immunology, Molecular biology

## Abstract

Immunosenescence, the gradual deterioration of the immune system with age, reduces the efficacy of immune checkpoint inhibitors (ICI) in cancer management. Although ICI offer promising survival benefits for advanced renal cell carcinoma (aRCC), their effectiveness across different age groups remains poorly understood. This study aimed to evaluate age-related survival outcomes in aRCC patients receiving ICI using integrated cohorts. Using data from the Surveillance, Epidemiology, and End Results (SEER) program (2004–2021), we identified patients with aRCC across the pre-ICI and ICI eras and stratified them into younger (<65 years) and older ($\ge$65 years) groups. Survival analyses, including Kaplan-Meier curves and multivariate Cox regression models, were performed to assess overall survival (OS). Key prognostic factors, such as tumor grade, surgical intervention, and metastatic status, were analyzed using nomograms and receiver operating characteristic (ROC) curves for predictive accuracy. A systematic search of PubMed, Web of Science, and Scopus also identified ten randomized controlled trials (RCTs) that met the meta-analysis criteria. Odds ratios (ORs) were calculated to evaluate age-stratified survival outcomes. Finally, laboratory-based immunohistochemistry (IHC) analysis of TNFSF15, an immune-related biomarker, was performed to explore molecular age-related differences in a hospital cohort. The SEER cohort included 21,904 patients (11,814 in the pre-ICI era and 10,090 in the ICI era). Age showed no significant impact on survival in the pre-ICI era (HR 1.050; 95% CI 0.97–1.32; p=0.203), while younger patients demonstrated superior outcomes in the ICI era (median OS, 16 vs. 13 months; HR, 1.37; 95% CI 1.24–1.51; p = 0.0001). Meta-analysis of 8,434 patients (4,207 ICI group, 4,227 control group) revealed significantly improved survival in younger patients receiving ICI (pooled OR 0.76; 95% CI 0.64–0.89; p<0.0001), whereas the non-ICI cohort showed no significant age-dependent effects (pooled OR 0.93; 95% CI 0.79–1.09; p = 0.37).When comparing ICI versus control treatments, younger patients derived greater benefit (HR 0.69 [0.62, 0.77]) than older patients (HR 0.84 [0.74, 0.96]) p<0.0001). TNFSF15 expression demonstrated a significant negative correlation with age (Spearman correlation = − 0.6, p = 0.0001), with significantly higher expression in patients aged <65 years to those $\ge$65 years (p=0.0001). This comprehensive analysis demonstrates the superior efficacy of ICI in younger aRCC patients across multiple cohorts, supporting the development of age-stratified therapeutic approaches. Age-dependent expression of TNFSF15 suggests potential molecular mechanisms underlying differential treatment responses.

## Introduction

Kidney cancer is expected to affect an estimated 81,800 new individuals and cause 14,890 deaths in the United States by 2023, making it a significant public health concern, particularly among older adults^1,2^. Renal cell carcinoma (RCCs) accounts for the majority of kidney cancer cases, with clear cell RCC (ccRCC) being the prevailing subtype^3^. Approximately 15-20% of patients are diagnosed with metastatic disease at the initial presentation, which significantly affects survival outcomes^4,5^. The higher incidence of RCC among older patients, who typically have a worse prognosis than younger individuals, emphasizes the urgent need for effective and well-tolerated systemic therapies. Historically, treatments for advanced RCC (aRCC) have been associated with substantial side effects and have shown limited improvements in survival^6,7^.

The treatment landscape for aRCC has evolved significantly with the advent of immune checkpoint inhibitors (ICI). Before 2005, treatment options were limited to surgery and cytokine-based therapies such as high-dose interleukin-2 (IL-2) and interferon-alpha (IFN-$\alpha$)^8^. Targeted therapies, particularly vascular endothelial growth factor (VEGF) inhibitors, tyrosine kinase inhibitors (TKIs), and mammalian target of rapamycin (mTOR) inhibitors, have provided new options for patients with metastatic disease^8^.

A pivotal moment in RCC management came in November 2015, with the FDA approval of nivolumab, an anti-PD-1 therapy, for second-line treatment^9,10^. Subsequently, various ICI-based combinations have demonstrated significant improvements in overall survival (OS), progression-free survival (PFS), and overall response rate (ORR) compared to traditional therapies^11,12^. Currently, first-line treatment options for metastatic RCC include dual ICI combinations or ICI combined with TKIs^13–20^. Treatment selection should be guided by the International Metastatic RCC Database Consortium (IMDC) risk criteria^21^. For patients with kidney-in-place and favorable- or intermediate-risk disease, cytoreductive nephrectomy may be considered. Post-nephrectomy patients who are asymptomatic with low disease burden may be candidates for initial active surveillance. Systemic therapy choices vary by risk group: favorable-risk patients may receive an ICI-VEGFR TKI combination, while intermediate- or poor-risk patients should be considered for doublet regimens. In select cases, monotherapy with either an ICI or VEGFR TKI may be appropriate based on individual comorbidities^21,22^. Despite these advances, the relative efficacy of different ICI combinations remains unclear due to the absence of direct phase III randomized controlled trials (RCTs) comparing these approaches. This gap in evidence highlights the need for further research to optimize treatment selection for individual patients^23–25^.

ICI established as critical components in treating many cancers, including aRCC, contribute to both curative and palliative care^26,27^. Age-related variations in treatment response have been observed, which may be influenced by immunosenescence - changes in immune system function that occur with aging^28,29^. These age-associated changes include alterations in hematopoiesis with a shift toward myeloid cell development, decreased lymphopoiesis, modifications in T-cell populations, and reduced production of co-stimulatory molecules^30–33^. While these biological changes could potentially influence treatment outcomes, the direct relationship between immunosenescence and ICI efficacy requires further investigation. Current evidence examining age-related treatment responses to ICI is primarily derived from small case series and brief reports, highlighting the need for more comprehensive studies to better understand the impact of age on immunotherapy effectiveness^34–37^.

Given the limited scope of previous research, our study aimed to address these gaps by conducting an extensive population-based analysis using data from the SEER database, alongside a meta-analysis, to examine oncologic outcomes in aRCC patients receiving ICI therapies across different age groups. Specifically, we compared overall survival on ICI therapy between younger (<65 years) and older ($\ge$65 years) patients, and investigated immune-related biomarkers associated with age in ccRCC patients. Our goal is to provide a comprehensive analysis of age-related differences in outcomes among patients receiving ICI for aRCC, contributing to the development of more informed treatment strategies.

## Results

### SEER analysis

#### Cohort characteristics and chi-square analysis by age group in ICI vs. non-ICI eras

**Fig. 1:**
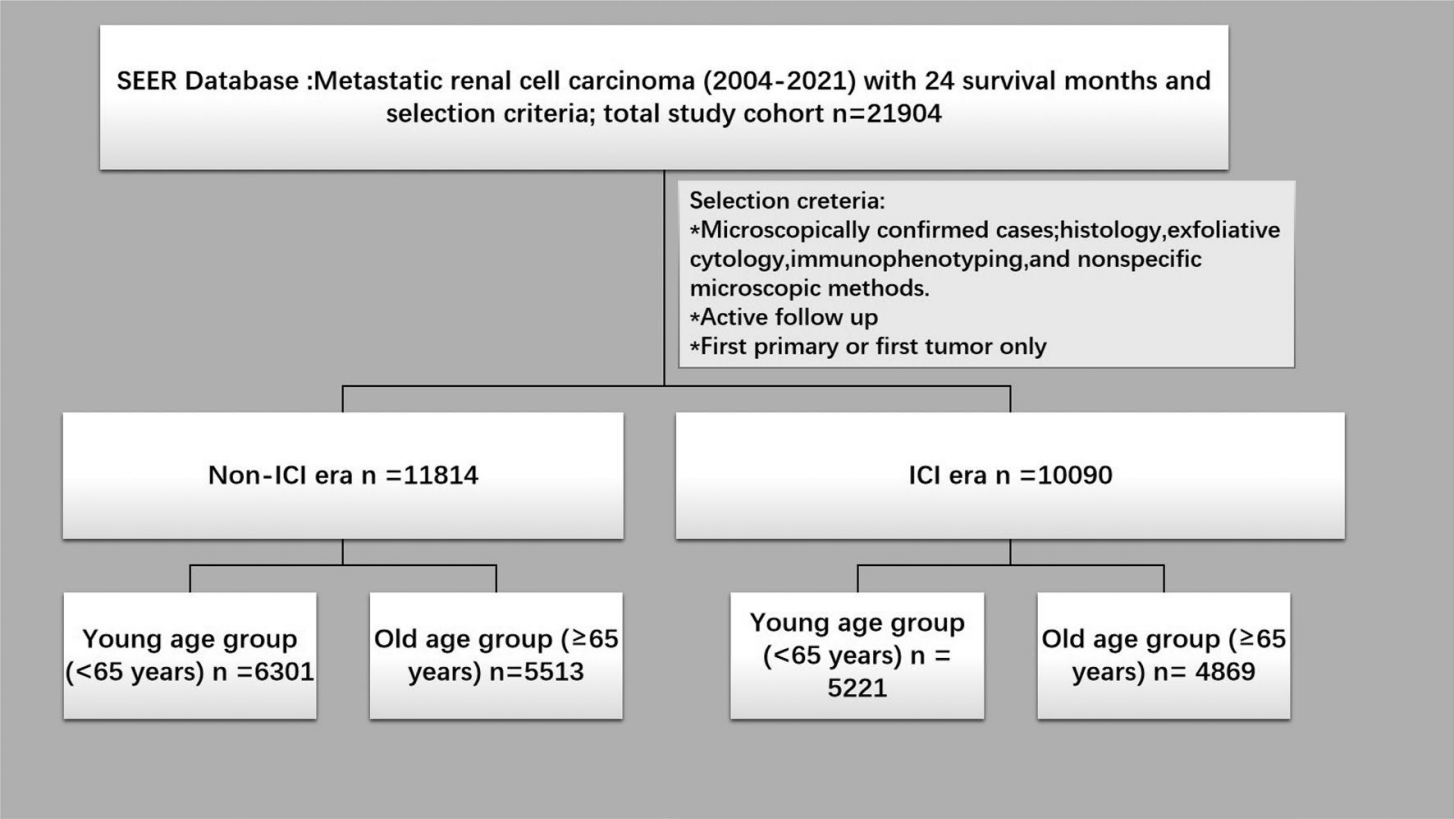
SEER database flowchart shows the detailed description of the inclusion criteria for advanced renal cell carcinoma patients in both ICI and Non-ICI eras.

A cohort of 21,904 patients diagnosed with aRCC has been extracted from the SEER database, including 11,814 cases reported before the advent of immune-checkpoint inhibitors (non-ICI period) and 10,090 cases recorded during the ICI era (Fig. 1). The primary demographic characteristics of patients over both eras were white, married males with either poor or undifferentiated tumor grades, larger tumor volumes, and no metastatic involvement in the bone, brain, or liver. A comparative examination of study variables using the chi-square test between the non-ICI period and the ICI era demonstrated statistically significant differences across the majority of variables (p < 0.05) among both age groups. No statistically significant variations were seen in marital status and sex, p > 0.05. Table 1 provides a concise overview of the clinico-pathological characteristics and multi-modal therapeutic approaches of individuals diagnosed with aRCC.

**Table 1:** Characteristics of Advanced Renal Cell Carcinoma Patients Across Both Age Groups in the ICI and Non-ICI Eras.

Parameters	Young group (<65 years)		p-value	Old group ($$\ge$$65 years)		p-value
	Non-ICI Count (%)	ICI Count (%)		Non-ICI Count (%)	ICI Count (%)	
Total	6301 (100)	5221 (100)		5513 (100)	4869 (100)	
Sex			0.645			<0.001
Male	4532 (71.93)	3776 (72.32)		3495 (63.40)	3262 (67.00)	
Female	1769 (28.07)	1445 (27.68)		2018 (36.60)	1607 (33.00)	
Surgery			<0.001			<0.001
Yes	3313 (52.58)	3116 (59.68)		2460 (44.62)	2573 (52.84)	
No	2988 (47.42)	2105 (40.32)		3053 (55.38)	2296 (47.16)	
Radiation			<0.001			<0.001
Yes	1961 (31.12)	1042 (19.96)		1261 (22.87)	788 (16.18)	
No	4340 (68.88)	4179 (80.04)		4252 (77.13)	4081 (83.82)	
Chemotherapy			<0.001			<0.001
Yes	3077 (48.83)	1535 (29.4)		2005 (36.37)	1224 (25.14)	
No	3224 (51.17)	3686 (70.6)		3508 (63.63)	3645 (74.86)	
Lung metastasis			<0.001			<0.001
Yes	1676 (26.6)	1852 (35.47)		1309 (23.74)	1695 (34.81)	
No	1487 (23.6)	3314 (63.47)		1538 (27.90)	3112 (63.91)	
Bone metastasis			<0.001			<0.001
Yes	1123 (17.82)	1235 (23.65)		935 (16.96)	1238 (25.43)	
No	2065 (32.77)	3947 (75.6)		1932 (35.04)	3584 (73.61)	
Brain metastasis			<0.001			<0.001
Yes	421 (6.68)	456 (8.73)		242 (4.39)	306 (6.28)	
No	2738 (43.45)	4715 (90.31)		2607 (47.29)	4498 (92.38)	
Liver metastasis			<0.001			<0.001
Yes	579 (9.19)	631 (12.09)		454 (8.24)	523 (10.74)	
No	2573 (40.83)	4534 (86.84)		2396 (43.46)	4292 (88.15)	
Laterality			<0.001			<0.001
Right	2864 (45.45)	2445 (46.83)		2554 (46.33)	2296 (47.16)	
Left	3180 (50.47)	2646 (50.68)		2690 (48.79)	2395 (49.19)	
Both side	257 (4.08)	130 (2.49)		269 (4.88)	178 (3.66)	
Race			<0.001			<0.001
White	5025 (79.75)	4208 (80.6)		4716 (85.54)	4095 (84.10)	
Black	802 (12.73)	477 (9.14)		412 (7.47)	364 (7.48)	
Others	474 (7.52)	536 (10.27)		385 (6.98)	410 (8.42)	
Marital status			0.645			<0.001
Yes	3604 (57.2)	2964 (56.77)		3264 (59.21)	2897 (59.50)	
Others	2697 (42.8)	2257 (43.23)		2249 (40.79)	1972 (40.50)	
Grade			<0.001			<0.001
Well, moderate	598 (9.49)	854 (16.36)		697 (12.64)	786 (16.14)	
Poor, undifferentiated	2928 (46.47)	1895 (36.3)		2074 (37.62)	1585 (32.55)	
Income			<0.001			<0.001
< $60,000	1379 (21.89)	805 (15.42)		1178 (21.4)	788 (16.2)	
> $60,000	4922 (78.11)	4416 (84.58)		4335 (78.6)	4081 (83.8)	
Tumor size			<0.001			<0.001
<4 cm	359 (5.7)	441 (8.45)		441 (8.0)	476 (9.8)	
4-7 cm	1008 (16.0)	1188 (22.75)		1301 (23.6)	1342 (27.6)	
>7 cm	4934 (78.31)	3592 (68.8)		3771 (68.41)	3051 (62.7)	

#### Survival analysis of age-related disparities in ICI vs. non-ICI eras

**Fig. 2:**
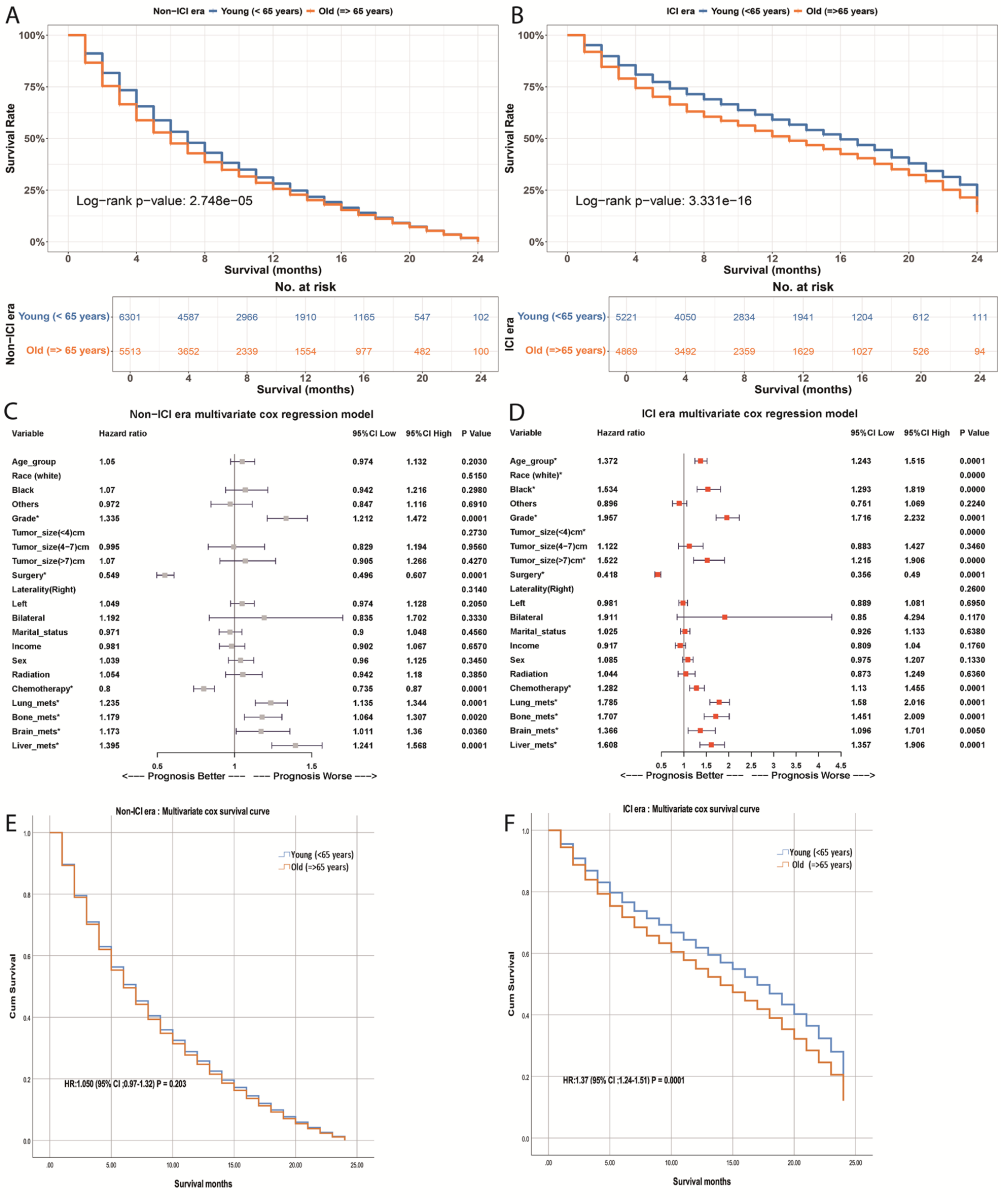
Age group survival differences in non-ICI and ICI eras. (**A**,**B**) Kaplan–Meier survival curves for younger (<65 years) vs. older ($\ge$65 years) in non-ICI (**A**) and ICI (**B**) eras. (**C**,**D**) Multivariate Cox models: in non-ICI (**C**), HR for $\ge$65 years is 1.05 (95% CI 0.97–1.13, p = 0.203); in ICI (**D**), HR is 1.37 (95% CI 1.24–1.51, p = 0.0001). (**E**,**F**) Cox survival curves in non-ICI (**E**) and ICI (**F**) eras for both age groups.

Upon analyzing OS variations to assess disparities between age groups across both eras, we observed no statistically significant difference in survival rates between individuals aged < 65 years and those aged $\ge$65 years during the non-ICI era, with median survival rates of 7 months and 6 months, respectively. Multivariate analysis confirmed that age did not demonstrate a statistically significant difference in the non-ICI period (hazard ratio [HR]: 1.050; 95% confidence interval [CI]: 0.97–1.32; p = 0.203) (Fig. 2A,C,E, and Table 2).

**Table 2:** Survival Patterns of Advanced Renal Cell Carcinoma Patients Across Both Age Groups in the ICI and Non-ICI Eras.

Parameters	<65 years group			$$\ge$$65 years group		
	(median months)			(median months)		
	Non-ICI era	ICI era	Log rank	Non-ICI era	ICI era	Log rank
	(7 months)	(16 months)	P value	(6 months)	(13 months)	P value
Sex						
Male	7	17	0.0001	6	14	0.0001
Female	7	15	0.0001	6	11	0.0001
Race						
White	7	17	0.0001	6	13	0.0001
Black	6	11	0.0001	6	9	0.0001
Others	8	18	0.0001	6	16	0.0001
Marital status						
Yes	7	18	0.0001	6	14	0.0001
Others	7	14	0.0001	6	11	0.0001
Laterality						
Both side	5	6	0.49	4	4	0.135
Right side	7	17	0.0001	6	13	0.0001
Left side	7	16	0.0001	6	14	0.0001
Surgery status						
Performed	9	22	0.0001	10	21	0.0001
Others	5	7	0.0001	4	6	0.0001
Radiation status						
Yes	6	10	0.0001	5	7	0.0001
No	7	19	0.0001	6	16	0.0001
Chemotherapy						
Yes	8	11	0.0001	7	10	0.0001
No	6	20	0.0001	5	16	0.0001
Metastatic site						
Bone						
Yes	6	8	0.0001	4	6	0.0001
No	8	20	0.0001	7	17	0.0001
Brain						
Yes	5	6	0.0001	4	4	0.0001
No	8	18	0.0001	7	14	0.0001
Liver						
Yes	5	5	0.0001	4	4	0.0001
No	8	18	0.0001	7	15	0.0001
Lung						
Yes	6	9	0.0001	5	6	0.0001
No	9	21	0.0001	8	19	0.0001
Grade						
Well, moderate	10	17	0.0001	9	23	0.0001
Poor, undifferentiated	8	20	0.0001	8	19	0.0001
Income						
< $60,000	7	15	0.0001	6	11	0.0001
> $60,000	7	17	0.0001	6	13	0.0001
Tumor size						
< 4 cm	7	21	0.0001	6	17	0.0001
4-7 cm	8	20	0.0001	6	18	0.0001
>7 cm	7	14	0.0001	6	11	0.0001

In contrast, noteworthy differences were observed among two age groups during the ICI era, with median survival rates of 16 months for the younger group compared to 13 months for the older group. Even after adjusting for significant variables identified in univariate analysis, age remained an independent risk factor in the ICI era, with an HR of 1.37 (95% CI 1.24–1.51; p = 0.0001) (Fig. 2B,D,F, Table 2).

Further evaluation of OS differences between the non-ICI and ICI eras revealed that individuals in the ICI era had a significantly higher chance of survival in both age groups (p = 0.0001; Fig. S1). Additionally, in both age groups (<65 years vs. $\ge$65 years), we found that the median survival duration for all cohort variables including basic characteristics, multimodal therapies, and metastatic status increased during the ICI period (p < 0.05; Table 2).

**Fig. 3:**
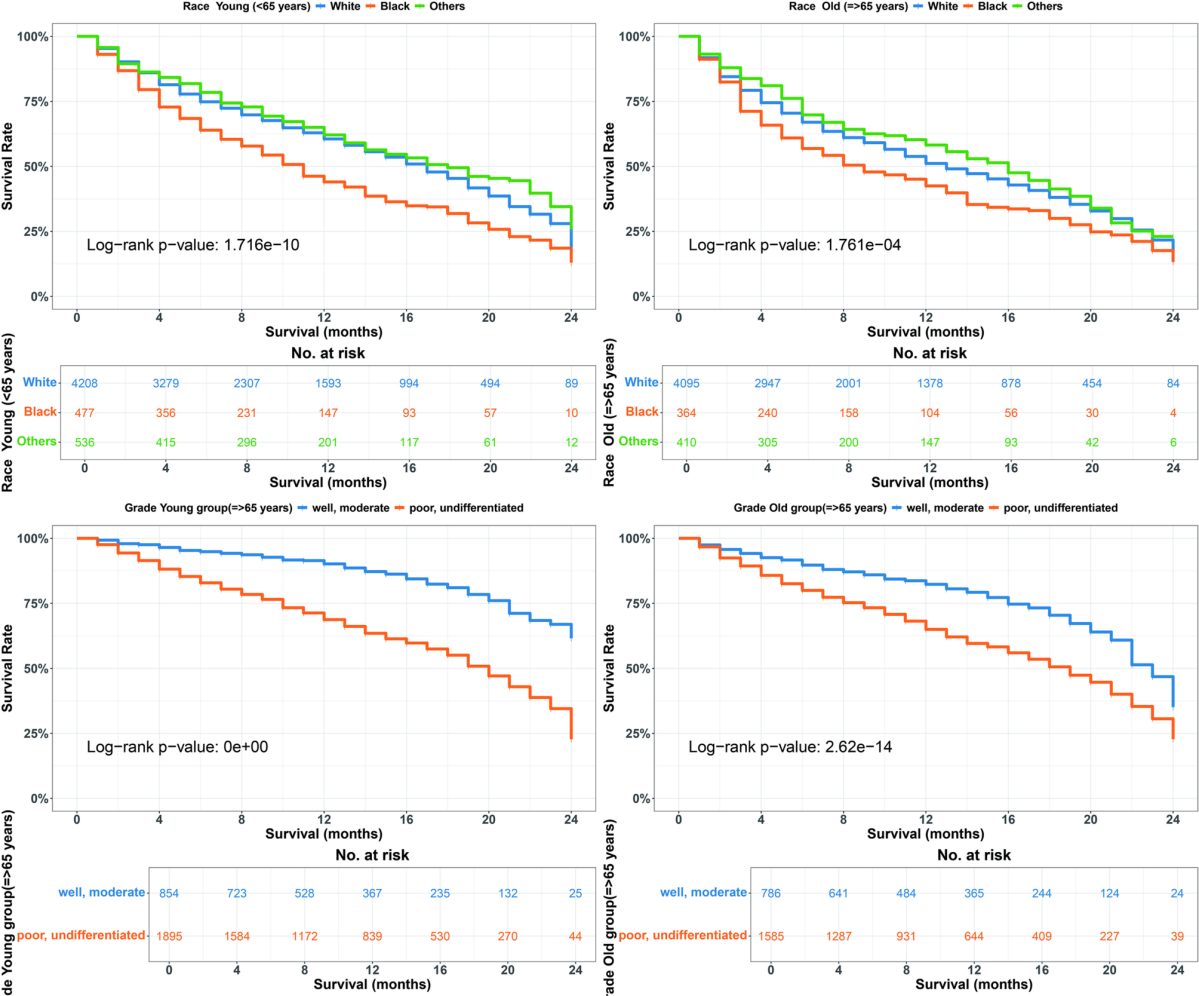
Overall survival (OS) differences by race and tumor grade in < 65years and $\ge$65 years age groups during the ICI era. Top: OS by race (White, Black, Others). Bottom: OS by tumor grade (well/moderate vs. poor/undifferentiated).

**Fig. 4:**
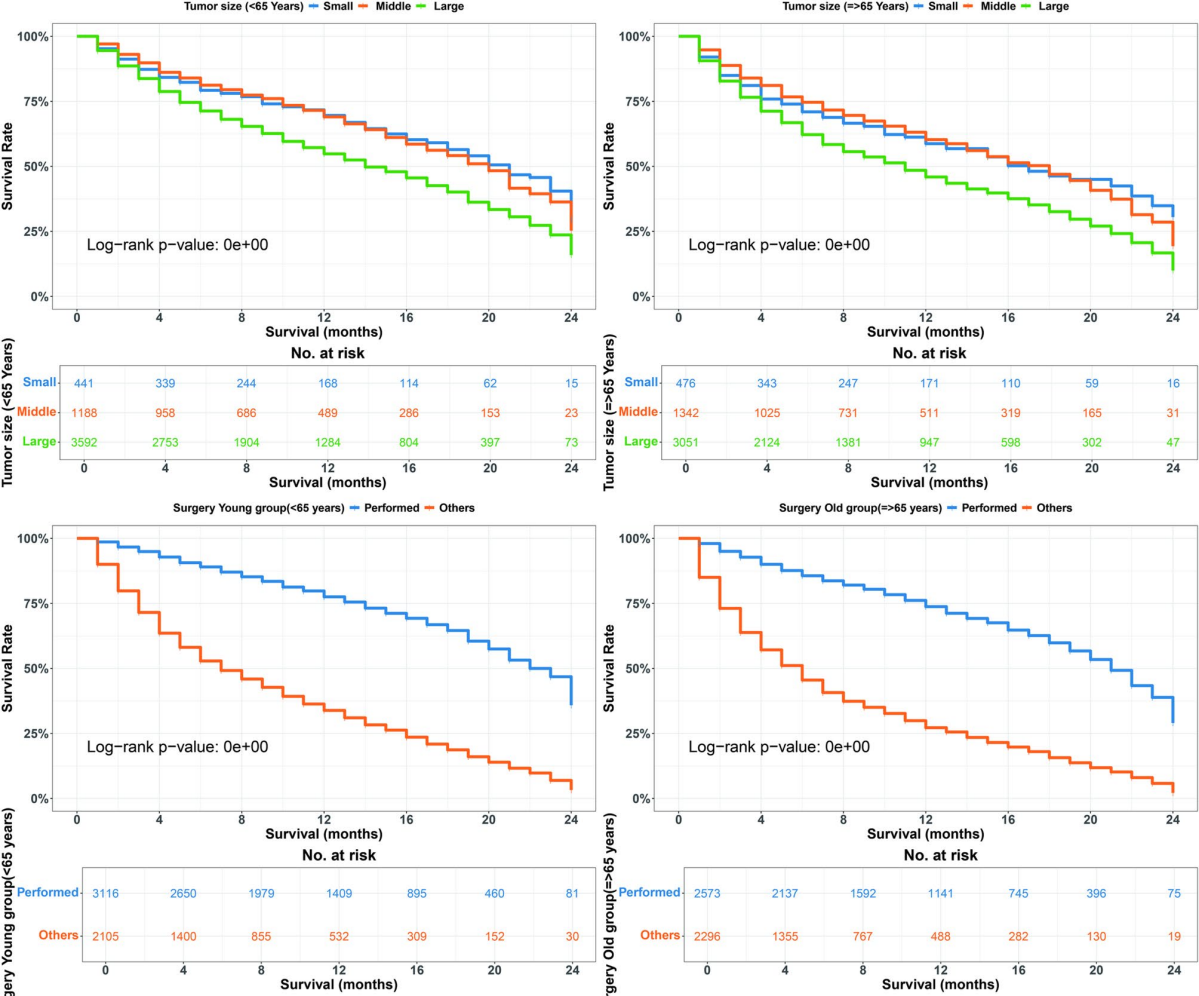
OS differences by tumor size and surgery in <65years and $\ge$65 years age groups during the ICI era. Top: OS by tumor size (< 4 cm, 4–7 cm, > 7 cm). Bottom: OS by surgical status (Performed vs. No).

Our analysis of OS variations within the group receiving immune checkpoint inhibitors indicates that black individuals, those with large tumors, poor or undifferentiated grades, and those who did not undergo surgery demonstrated significantly poorer survival outcomes across all age groups (p < 0.05, Figs. 3,  4). While surgical intervention improved survival compared to non-surgical treatment (22 vs. 7 months in younger patients; 21 vs. 6 months in older patients) in the ICI era. However, results should be interpreted cautiously as SEER doesn’t differentiate between curative nephrectomy for localized disease and cytoreductive nephrectomy for metastatic disease. The association likely partly reflects the selection of patients with better disease characteristics for surgery, as shown in CARMENA and SURTIME trials^38,39^.

**Fig. 5:**
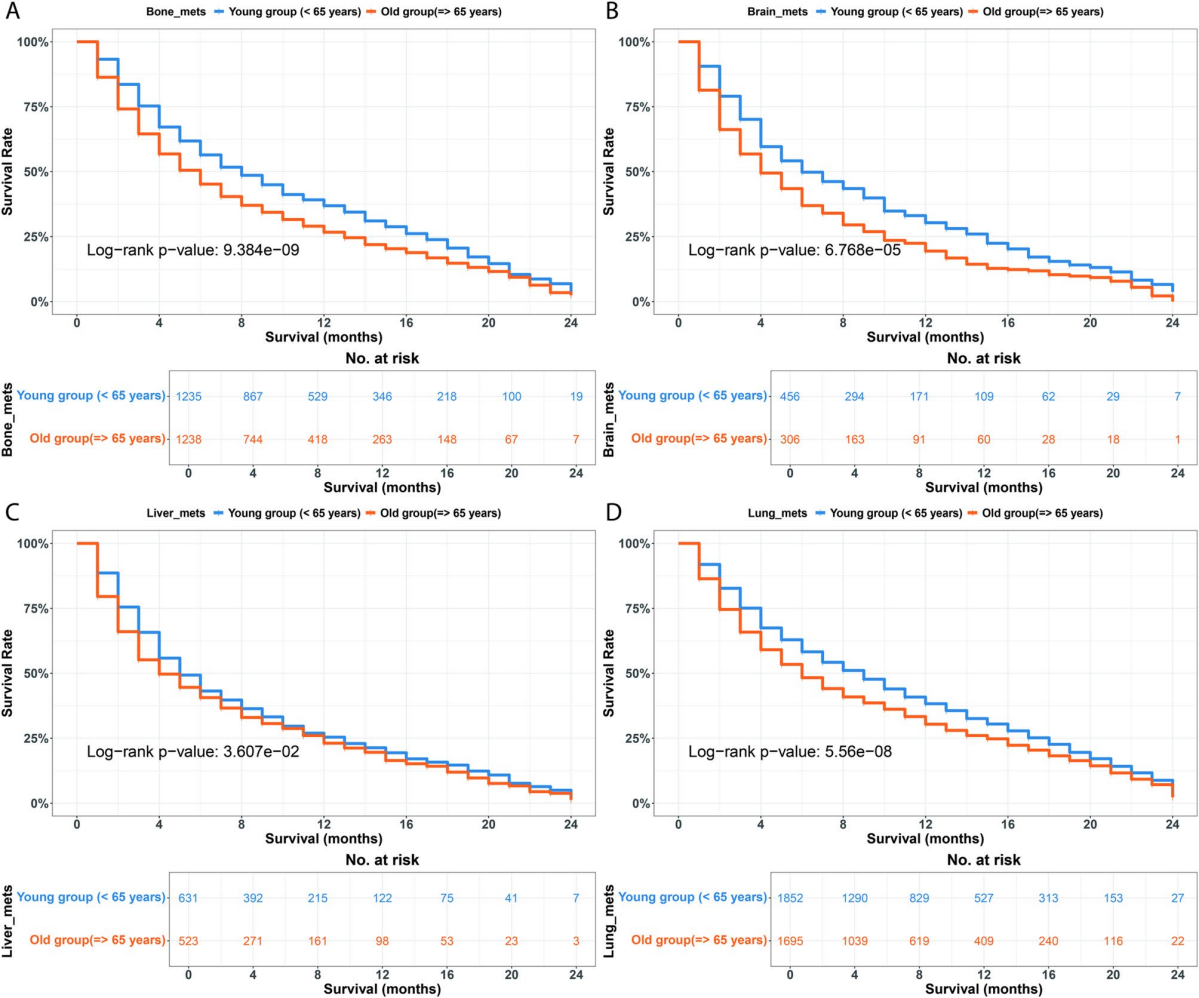
Overall survival (OS) differences between < 65 years vs. $\ge$65 years age groups according to metastatic site during ICI era. (**A**) Bone. (**B**) Brain. (**C**) Liver. (**D**) Lung.

The investigation into patient survival among individuals with and without metastases in bone, brain, liver, and lung during the immunotherapy era revealed that those with metastases exhibited a shorter life expectancy compared to their counterparts without metastases, irrespective of age group (young or old) Fig. S2. A comparison of survival status for patients with metastasis across various age groups and metastatic sites during the ICI era indicated significant disparities. The older age group with metastases to the bone, brain, liver, and lung demonstrated significantly lower survival rates compared to the younger group (p < 0.05; Fig. 5).

Analysis of metastatic locations within the ICI category revealed significant differences in the young cohort, with a log-rank p-value of 3.054e$^{-10}$, indicating that liver metastasis correlated with the lowest median survival (median = 5 months). In the older group, survival exhibited significant differences, indicated by a log-rank p-value of 2.908e$^{-07}$, with both brain and liver metastases associated with the lowest median survival of 4 months. Lung metastasis exhibited the highest survival rates relative to other metastasis types in both age groups, with a median survival of 9 months for individuals under 65 years and 6 months for those aged 65 and older (Fig. S3, and Table 2).

#### Logistic regression model for factors affecting survival across age groups in ICI era

Univariate analysis revealed significant associations between certain clinical and demographic factors and survival outcomes in the ICI era. Among both younger and older age groups, black race, unmarried status, poor or undifferentiated tumor grade, larger tumor size, absence of surgery, and receipt of radiation therapy were linked to decreased survival rates (p < 0.05).

Multivariate analysis further validated these findings. In the younger cohort (<65 years), black patients (HR 1.591, 95% CI = 1.264–2.001, p = 0.0001), those with poor or undifferentiated tumor grade (HR 2.270, 95% CI = 1.842–2.799, p = 0.0001), and those not undergoing surgery (HR 0.469, CI = 0.37-0.59, p = 0.0001) had significantly poorer survival. In the older cohort ($\ge$65 years), similar trends were observed, with black race (HR 1.539, 95% CI = 1.188–1.996, p = 0.0001), poor tumor grade (HR 1.779, 95% CI = 1.499–2.111, p = 0.0001), tumor size >7 cm (HR 1.849, 95% CI = 1.336–2.559, p = 0.0001), and lack of surgery (HR 0.38, CI = 0.30-0.47, p = 0.0001) associated with worse survival outcomes.

**Fig. 6:**
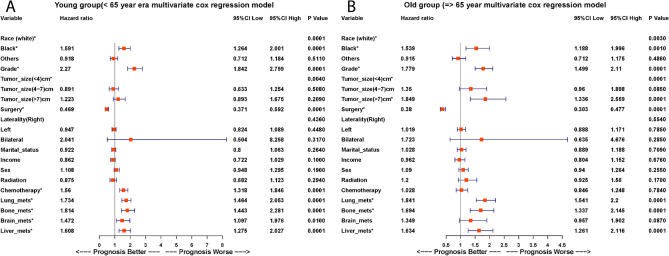
Multivariate Cox regression for prognostic factors during the ICI era. (**A**) Younger group: Significant predictors include black race (HR 1.591, CI = 1.264-2.001, p = 0.0001), tumor grade (HR 2.27, CI = 1.842-2.799, p = 0.0001), and surgery (HR 0.469, CI = 0.37-0.59, p = 0.0001). (**B**) Older group: Significant predictors include black race (HR 1.539, CI = 1.188-1.996, p = 0.001), tumor grade (HR 1.779, CI = 1.499-2.111, p = 0.0001), and surgery (HR 0.38, CI = 0.30-0.47, p = 0.0001). Metastatic sites (lung, bone, brain, liver) show significant OS associations in both groups.

Both univariate and multivariate analyses consistently demonstrated that liver, lung, and bone metastases were significantly associated with decreased survival in both age groups (p = 0.0001). Notably, brain metastases in the older cohort showed no significant difference in survival (HR 0.741, 95% CI = 0.526–1.045, p = 0.087) compared to patients without brain metastases (Fig. 6A,B and Table S1, S2).

#### Prognostic nomogram model and ROC validation for age groups in ICI era

**Fig. 7:**
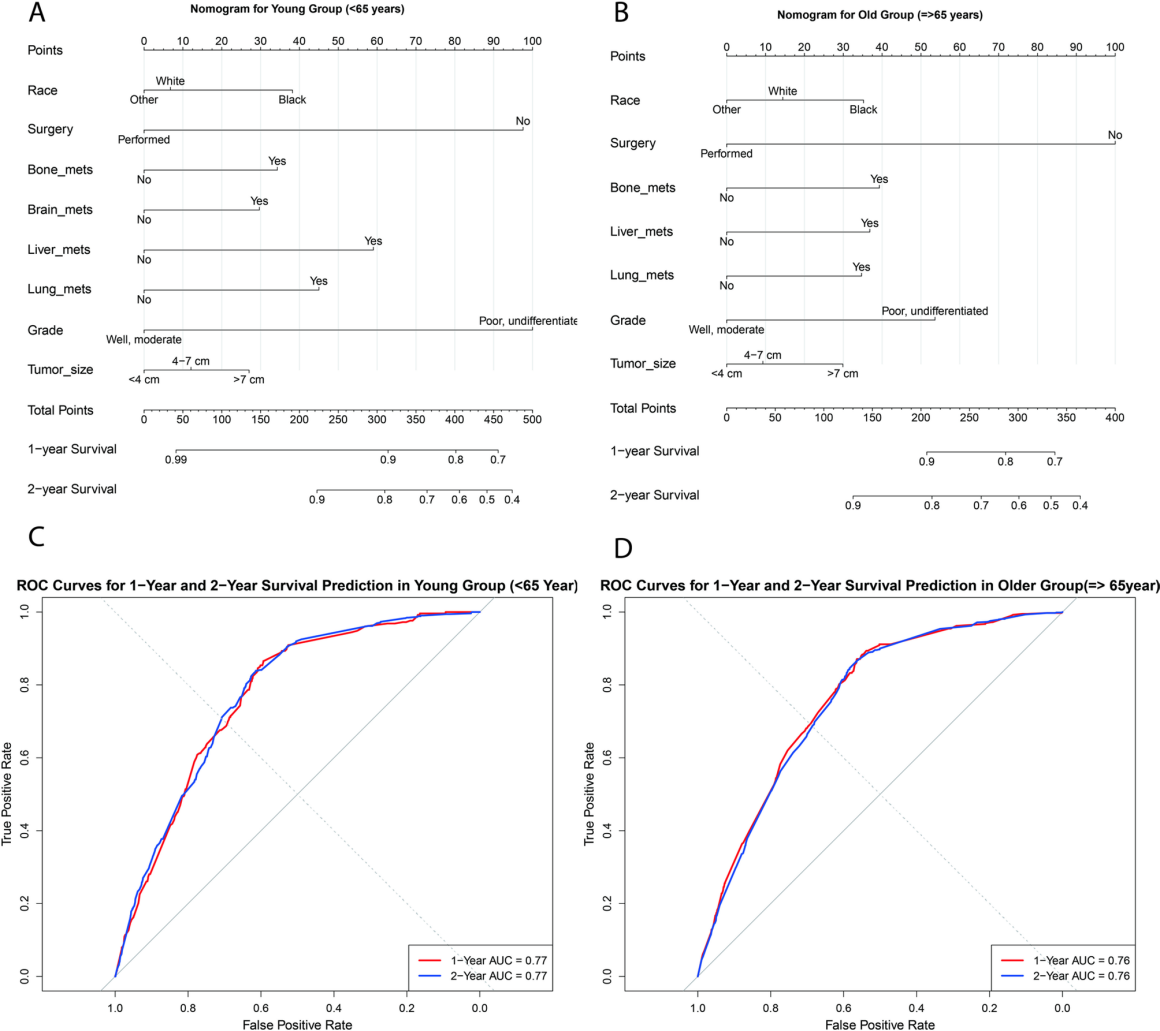
(**A**,**B**). Nomogram prediction model for 1- and 2-year survival based on significant variables in each age group during the ICI era, with concordance indices (C-index) of 0.75 for the younger group and 0.74 for the older group. (**C**,**D**) Receiver Operating Characteristic (ROC) curves evaluating the predictive accuracy of the nomogram at 1 and 2 years for both age groups in the ICI era.

A prognostic nomogram model was constructed using key survival predictors identified via multivariate Cox regression analysis for aRCC in both age groups during the ICI era. Prognostic variables were weighted and assigned scores, with cumulative scores used to estimate 1- and 2-year survival probabilities. For the ICI-treated cohorts, the nomogram for OS demonstrated strong predictive performance, with a concordance index (C-index) of 0.75 for patients under 65 and 0.74 for those 65 and older. Pathological grade and surgical intervention were the most significant factors influencing prognosis, followed by metastatic status (Fig. 7A,B).

Receiver operating characteristic (ROC) curves were generated at 1- and 2-year intervals to further assess model accuracy. The area under the ROC curve (AUC) values, indicating high predictive accuracy, were 77% for the <65 age group and 76% for the $\ge$65 age group over a 2-year period, as illustrated in Fig. 7C,D.

#### Chronological age impact on survival in ICI era: a stratified analysis of three age groups

In addition to our primary study design comparing younger and older populations, we further stratified participants to examine the effect of chronological age on OS across three specific cohorts: younger individuals (<65 years), those aged 65–74 years, and the elderly group (>74 years). KM survival analysis revealed a significantly poorer prognosis in the elderly cohort (p = 0.0001), indicating a clear survival disparity among age groups. Additionally, multivariate logistic analysis confirmed that advanced age in the elderly group was an independent prognostic factor for OS, with HR of 1.678 (95% CI 1.46–1.92, p = 0.0001) (Fig. S4).

### Meta-analysis

#### Study selection and characteristics

Our study followed PRISMA guidelines, and we have made selection for articles and summarized it in the PRISMA flow chart. Ultimately, 10 RCTs involving 8,434 patients were identified as suitable to be included in this meta-analysis. The demographic characteristics and oncological outcomes for each study are presented in Table 3. Although our primary focus was on advanced/metastatic RCC, we included adjuvant trials in for advanced localized RCC to provide a comprehensive assessment of age-related outcomes across the disease spectrum. We analyzed and reported these results separately to avoid inappropriate comparisons between metastatic and adjuvant settings, recognizing their distinct clinical contexts with different treatment goals and expected outcomes. Across these trials, the aRCC patient median age was from (58-64 years). The proportion of younger patients (<65 years) ranged from 55% to 73%, while older patients ($\ge$65 years) accounted for 27% to 45% of the total participants across studies.

**Fig. 8:**
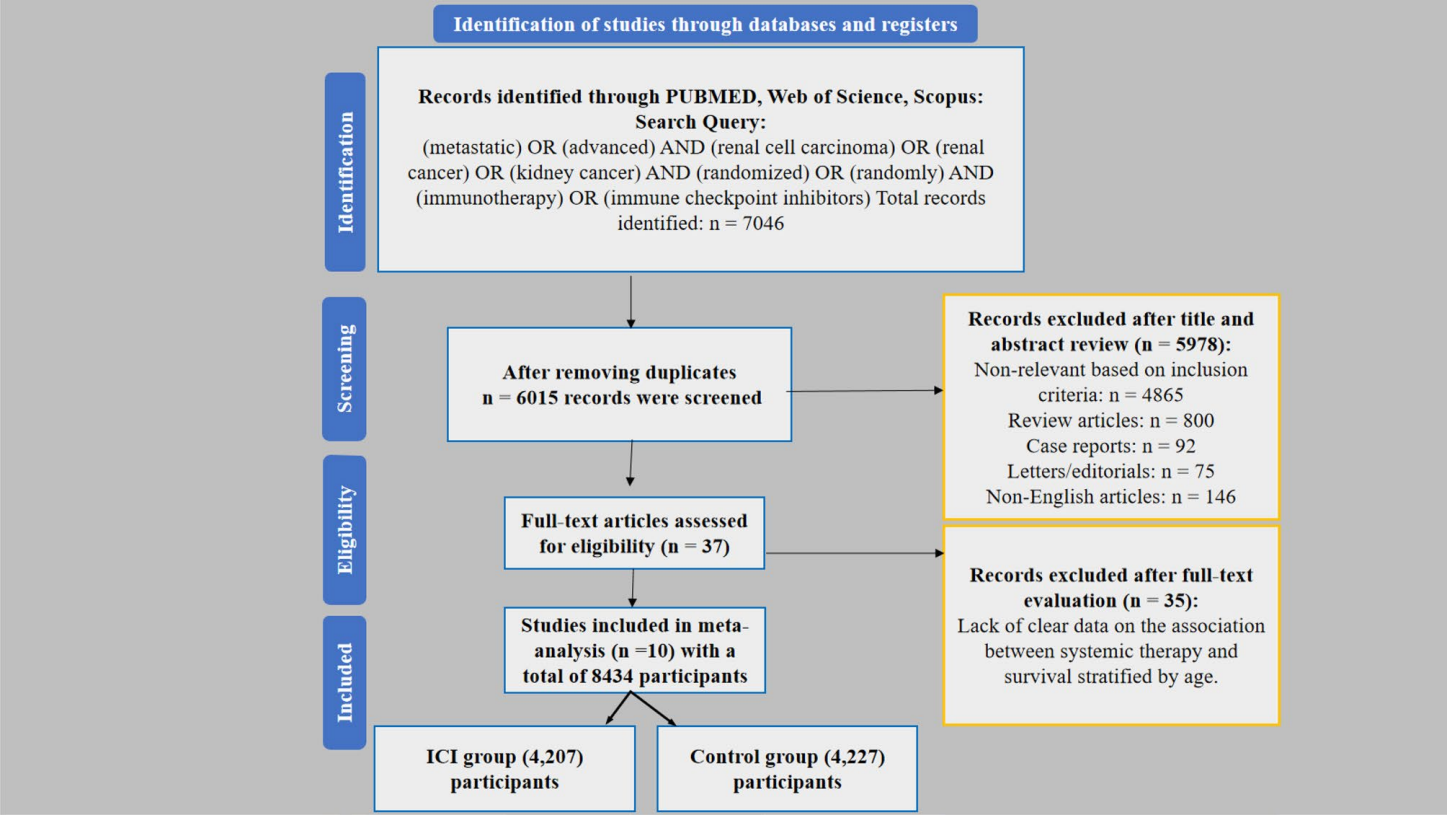
PRISMA flow diagram outlining the article selection process for this systematic review and meta-analysis on advanced renal cell carcinoma (aRCC) receiving ICI therapy.

Of the total cohort of 8,434 patients, 3,864 were older than 65 years (45.8%). In the ICI treatment group (4,207 patients), 1,918 (45.6%) were aged $\ge$65 years, while in the control group (4,227 patients), 1,946 (46.0%) were aged $\ge$65 years. The risk of bias for each domain across the included studies is outlined in (Fig. 8).

**Fig. 9:**
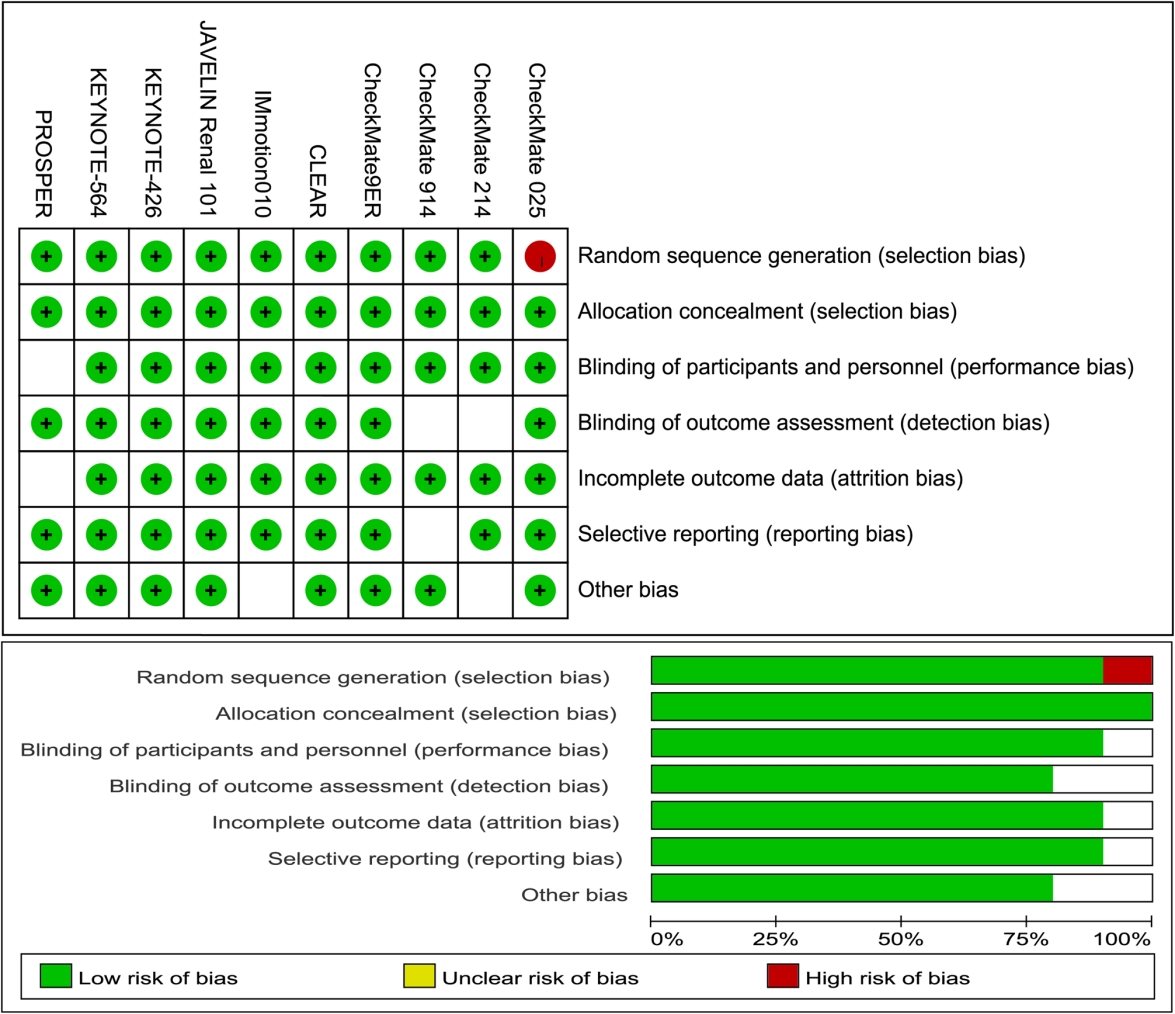
Risk-of-bias assessment of included studies on advanced renal cell carcinoma (aRCC), conducted using RevMan 5.1.4. The summary plot provides a visual overview of the proportion of studies with low, unclear, and high risks of bias in each domain.

All phase III RCTs that met the inclusion criteria were found to have either a low risk of bias or no significant concerns Fig. 9.

#### Age-dependent treatment effects in ICI versus non-ICI groups

Our meta-analysis employed two complementary statistical approaches to comprehensively assess age-related treatment effects. To compare survival outcomes between younger (<65 years) and older ($\ge$65 years) patients within each treatment group (ICI or non-ICI), we utilized odds ratios (ORs), allowing direct assessment of age-dependent efficacy patterns. For comparing treatment effects between ICI and non-ICI therapies in each age group, we employed hazard ratios (HRs), which account for both the occurrence of events and their timing. This dual methodological approach enabled us to examine both age-related differences within treatment modalities and treatment-related differences within age groups, providing a more comprehensive understanding of the interplay between age and ICI efficacy.

##### Overall survival in metastatic RCC

**Fig. 10:**
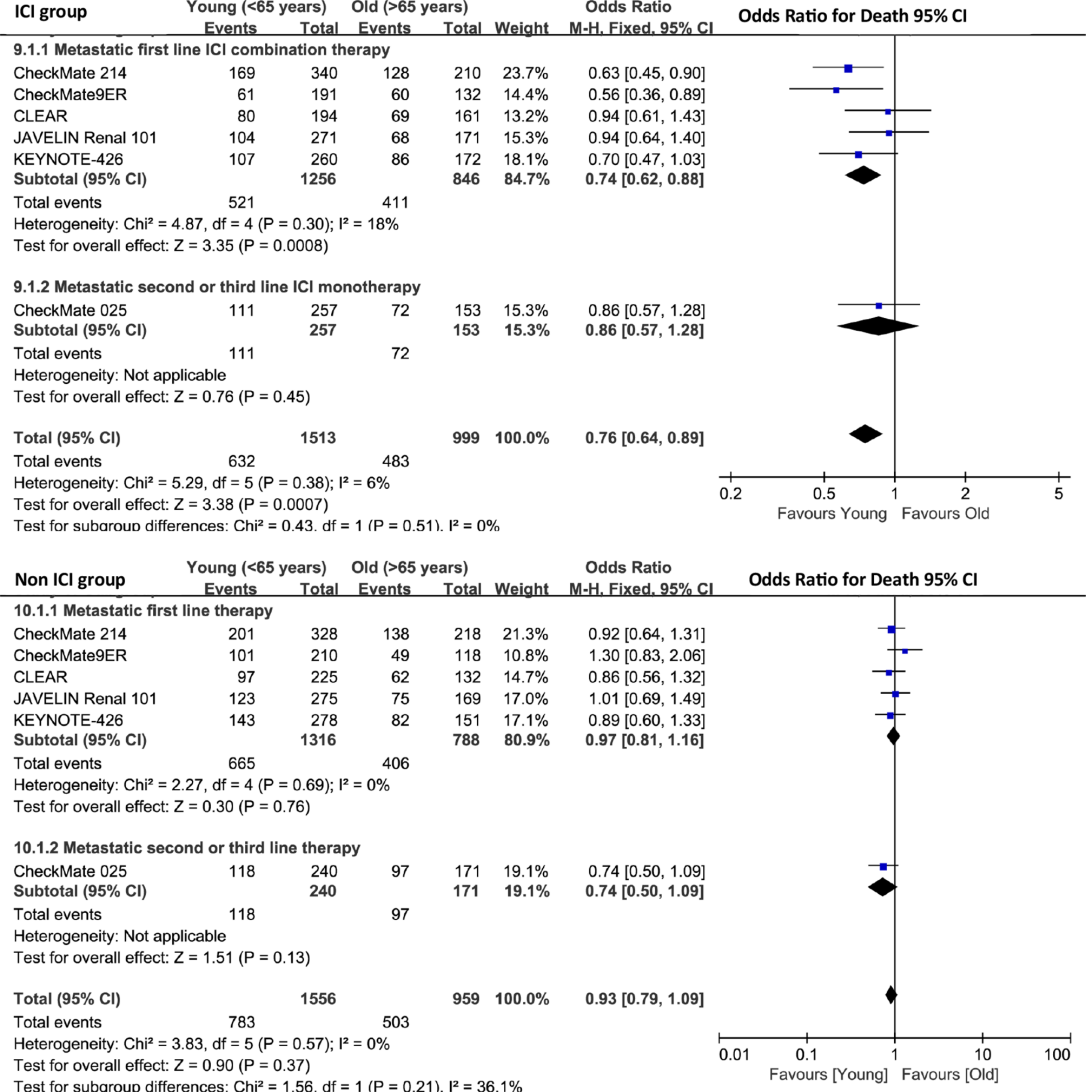
Meta-analysis of overall survival comparing ICI versus control treatments in younger (<65 years) versus older ($\ge$65 years) aRCC patients. The ICI group demonstrated significant survival advantages in first-line combination therapy (OR = 0.74, 95% CI 0.62–0.88, p = 0.0008), while second line no benifit(OR = 0.86, 95% CI 0.57–1.28, p = 0.45) with an overall mortality benefit (OR = 0.76, 95% CI 0.64–0.86, p < 0.0001). The control group showed non-significant age-related differences (overall OR = 0.93, 95% CI 0.79–1.02, p = 0.37), supporting superior efficacy of ICI-based treatment in younger patients.

In the ICI cohort, patients <65 years showed significantly improved overall survival compared to patients $\ge$65 years (pooled OR = 0.76; 95% CI 0.64–0.89; p < 0.0007) (Fig. 10). Subgroup analyses revealed distinct pattern across different treatment settings. First-line ICI combination therapies demonstrated significant benefits in CheckMate 214 (OR = 0.63; 95% CI 0.45–0.90) and CheckMate 9ER (OR = 0.56; 95% CI 0.36–0.89). The pooled analysis of first-line combination therapies yielded an OR of 0.74 (95% CI 0.62–0.88; p = 0.0008). Moderate heterogeneity was observed across ICI trials (Cochran’s Q = 5.29, p = 0.38; I$^{2} = 6\%$) (Fig. S5 A).

**Fig. 11:**
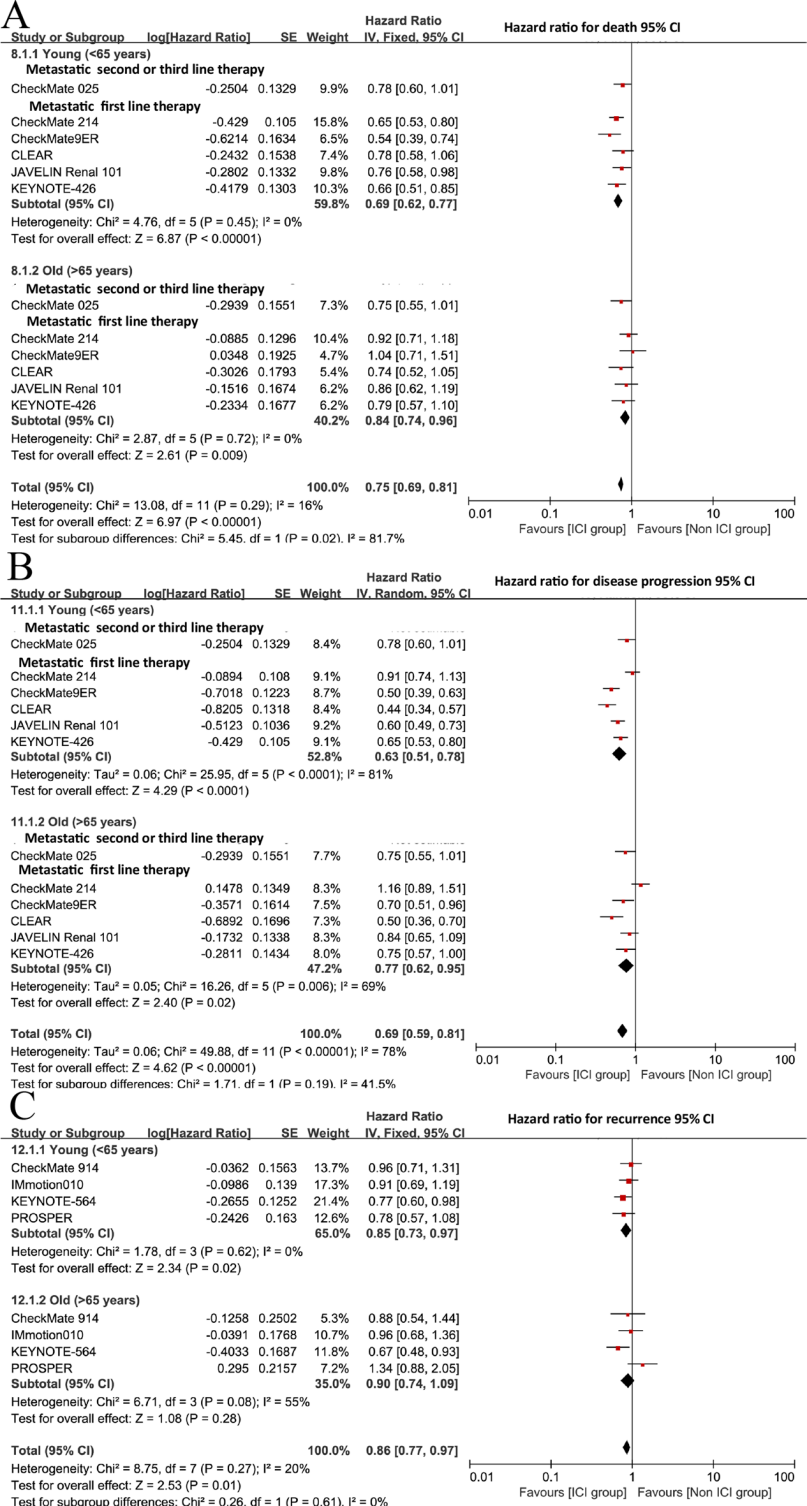
Meta-analysis of survival outcomes comparing ICI versus non-ICI treatments in younger (<65 years) versus older ($\ge$65 years) advanced renal cell carcinoma patients. (**A**) Overall survival: Hazard ratios (HR) for death show a significant survival benefit with ICI treatment. For younger patients (<65 years), the pooled HR was 0.69 (95% CI 0.62-0.77, p < 0.0001), while for older patients ($\ge$65 years), it was 0.84 (95% CI 0.74-0.96, p = 0.009). The overall HR between young and old was 0.75 (95% CI 0.69-0.81, p < 0.0001). (**B**) Progression-free survival: HR for disease progression indicated benefits with ICI in younger patients (HR 0.63, 95% CI 0.51-0.78, p < 0.0001) and older patients (HR = 0.77, 95% CI 0.62-0.95, p = 0.006). The overall HR between young and old was 0.69 (95% CI 0.59-0.81, p < 0.0001). (**C**) Disease-free survival: HR for recurrence showed a significant benefit with ICI in younger patients (HR 0.86, 95% CI 0.73-0.97, p = 0.02) and a non-significant trend in older patients (HR 0.90, 95% CI 0.74-1.09, p = 0.28). Overall, ICI treatments consistently improved survival outcomes, with greater efficacy in younger patients.

In the non-ICI cohort, age-dependent treatment effects were not statistically significant (pooled OR = 0.93; 95% CI 0.79–1.09; p = 0.37) (Fig. 10). First-line conventional therapies showed minimal age-based variation (OR = 0.97; 95% CI 0.81–1.16), while second- and third-line treatments demonstrated non-significant effects (OR = 0.74; 95% CI 0.50–1.09). The non-ICI group exhibited lower heterogeneity(Cochran’s Q = 3.83, p = 0.57; I$^{2} = 0\%$) compared to the ICI group (Fig. S5 B). Our hazard ratio analysis further clarified the efficacy of ICI versus control treatments in metastatic RCC, with notable age-related differences. Across all patients, ICI therapy significantly improved survival compared to control treatments (pooled HR 0.75; 95% CI 0.69–0.81; p < 0.00001) (Fig. 11A).

##### Progression-free survival in metastatic RCC

Regarding Progression-Free Survival, the ICI cohort exhibited a non-significant trend favoring younger patients (OR = 0.85; 95% CI 0.59–1.22; p = 0.37), with CheckMate 214 indicating the most substantial advantage (OR = 0.47; 95% CI 0.32–0.69) (Fig. S6 A). In the non-ICI group, older patients had markedly superior outcomes (OR = 1.42; 95% CI 1.18–1.71; p = 0.005; I$^{2} = 48\%$), especially in KEYNOTE-426 (OR = 2.04; 95% CI 1.33–3.12) (Fig. S7 A). Our hazard ratio analysis provided more definitive insights into the PFS benefits of ICI versus control treatments across age groups. Across all patients, ICI therapy significantly reduced the risk of disease progression compared to control treatments (pooled HR 0.69; 95% CI 0.59–0.81; p < 0.00001) (Fig. 11B).

##### The objective response rate in metastatic RCC

The Objective Response Rate for first-line ICI combination therapy shown comparable efficacy across age demographics (OR = 1.02; 95% CI 0.68–1.53) .Moreover, second-line ICI monotherapy indicated a trend favoring younger individuals (OR = 0.74; 95% CI 0.47–1.16) (Fig. S6B) while, the non-ICI cohort exhibited negligible age-related variation (OR = 1.01; 95% CI 0.82–1.25; p = 0.90) (Fig. S7 B).

##### Disease-free survival in adjuvant setting

Disease-Free Survival in adjuvant ICI trials indicated a trend favoring younger patients (OR = 0.84; 95% CI 0.68–1.03; p = 0.10) (Fig. S6C), but non-ICI therapies had no significant age-dependent effects (OR = 0.92; 95% CI 0.72–1.17; p = 0.49) (Fig. S7C). We assessed publication bias in the meta-analysis of ICI and control treatment subgroups (S8 A-F) for 2ry outcome. Our hazard ratio analysis provided a more comprehensive assessment of the efficacy of adjuvant ICI therapy in preventing recurrence across age groups. Across all patients, adjuvant ICI therapy significantly reduced the risk of recurrence compared to control treatments (pooled HR 0.86; 95% CI 0.77–0.97; p = 0.01) (Fig. 11C).

#### Age-dependent treatment effects across ICI classes

##### First-line treatment outcomes

Age-stratified analyses of first-line treatments revealed distinct efficacy patterns across immunotherapy combinations (Table 3). CheckMate 9ER demonstrated that nivolumab plus cabozantinib significantly reduced the risk of death in younger patients (<65 years) by 46% (HR 0.54; 95% CI 0.39–0.74). However, no survival benefit was observed in older patients ($\ge$65 years) (HR 1.03; 95% CI 0.71–1.51). Similarly, progression-free survival (PFS) showed a 50% reduction in risk for younger patients (HR 0.50; 95% CI 0.39–0.63), while the benefit was less pronounced in older patients (HR 0.70; 95% CI 0.51–0.96).

In JAVELIN Renal 101, avelumab plus axitinib also showed greater efficacy in younger patients, with a 24% reduction in overall mortality (HR 0.76; 95% CI 0.58–0.98) and a 40% reduction in progression risk (HR 0.60; 95% CI 0.49–0.74). Although benefits were observed in older patients, they were less pronounced and did not reach statistical significance for OS (HR 0.86; 95% CI 0.62–1.19) or PFS (HR 0.84; 95% CI 0.65–1.09).

KEYNOTE-426 highlighted similar findings with pembrolizumab plus axitinib, achieving a 34% reduction in the risk of death for younger patients (HR 0.66; 95% CI 0.51–0.85). However, the survival benefit was diminished in older patients (HR 0.78; 95% CI 0.57–1.1). The PFS advantage was also more robust in younger patients (HR 0.65; 95% CI 0.53–0.80) compared to older ones (HR = 0.76; 95% CI 0.57–1.0).

In CheckMate 214, nivolumab plus ipilimumab showed a striking age-dependent effect. Younger patients experienced a 43% reduction in the risk of death (HR 0.57; 95% CI 0.44–0.76), but no significant OS benefit was observed in older patients (HR 0.99; 95% CI 0.72–1.37). Notably, PFS benefits were minimal across both age groups.

Conversely, CLEAR demonstrated consistent efficacy across age groups for pembrolizumab plus lenvatinib. Younger patients showed a 37% reduction in overall mortality (HR 0.63; 95% CI 0.41–0.95), comparable to older patients (HR 0.61; 95% CI 0.40–0.95). The PFS benefits were similarly robust, with HRs of 0.37 (95% CI 0.28–0.49) and 0.43 (95% CI 0.31–0.61) for younger and older patients, respectively.

##### Second-line treatment outcomes

The CheckMate 025 trial of nivolumab versus everolimus revealed that younger patients (<65 years) experienced a trend toward improved overall survival (HR 0.78; 95% CI 0.60–1.01). However, no OS benefit was observed in older patients (HR 1.21; 95% CI 0.89–1.64), highlighting a more age-dependent efficacy profile in the second-line setting.

##### Adjuvant treatment outcomes

In the adjuvant setting, KEYNOTE-564 showed that pembrolizumab improved disease-free survival (DFS) consistently across age groups. Younger patients (<65 years) had a 23% reduction in recurrence risk (HR 0.77; 95% CI 0.60–0.98), while older patients ($\ge$65 years) experienced a 33% reduction (HR 0.67; 95% CI 0.48–0.93).

In contrast, other trials failed to demonstrate significant DFS benefits. Neither CheckMate 914 (nivolumab plus ipilimumab), IMmotion010 (atezolizumab), nor PROSPER (nivolumab) showed meaningful efficacy in either younger or older patients, underscoring the limited benefits of these regimens in the adjuvant setting.

**Table 3:** Study demographics and outcomes of included RCTs of ICI for aRCC stratified by age groups.

Study name and first author	Year	Treatment arm	Control arm	Number of patients (T/C)	Median age (T/C)	$\ge$65 years n(%) (T/C)	IMDC Classification, n(%)			HR (95%CI)	HR (95%CI)
							Favorable	Intermediate	Poor	of OS	of PFS
							T/C	T/C	T/C	(T vs. C)	(T vs. C)
										Young/Old	Young/Old
*1st line Treatment*											
CheckMate9ER, Motzer et al.	2022	Nivolumab + Cabozantinib	Sunitinib	323/328	62 (IQR: 55-69) / 61 (IQR: 53-67)	132(41)/118(36)	74(23)/72(22)	188(58)/188(57)	61(19)/68(21)	0.54 (0.39-0.74)/1.03(0.71-1.51)	0.5 (0.39-0.63)/0.7(0.51-0.96)
JAVELIN Renal 101, Motzer et al.	2019-2023	Avelumab + Axitinib	Sunitinib	442/444	62 (range: 29-83) / 61 (range: 27-88)	171(39)/169(38)	52(19)/60(21)	180(67)/201(69)	33(12)/24(8.3)	0.76 (0.58-0.98)/0.86(0.62-1.19)	0.60 (0.49-0.74)/0.84(0.65-1.09)
KEYNOTE-426, Powles et al.	2023	Pembrolizumab + Axitinib	Sunitinib	432/429	62 (IQR: 55-68) / 61 (IQR: 53-68)	172(40)/151(35)	138(32)/131(31)	238(55)/246(57)	56(13)/52(12)	0.66 (0.51-0.85)/0.78(0.57-1.1)	0.65 (0.53-0.80)/0.76(0.57-1.0)
CheckMate 214, Motzer et al.	2018-2022	Nivolumab + Ipilimumab	Sunitinib	550/546	62 (range: 26-85) / 62 (range: 21-85)	210(38)/218(40)	125(23)/124(23)	334(61)/333(61)	91(17)/89(16)	0.57 (0.44-0.76)/0.99(0.72-1.37)	0.91 (0.74-1.13)/1.16(0.89-1.51)
CLEAR, Motzer et al.	2021-2024	Pembrolizumab + Lenvatinib	Sunitinib	355/357	64 (range: 34-88) / 61 (range: 29-82)	161(45)/132(37)	110(27)/124(35)	210(64)/192(54)	33(9.3)/37(10)	0.63 (0.41-0.95)/0.61(0.40-0.95)	0.37 (0.28-0.49)/0.43(0.31-0.61)
*2nd line Treatment*											
CheckMate 025, Motzer et al.*	2015-2020	Nivolumab	Everolimus	410/411	62 (range: 23-88)/62 (range: 18-86)	NA	145(35)/148(36)	201(49)/203(49)	64(16)/60(15)	0.78 (0.60-1.01)/1.21(0.89-1.64)	NA
*Adjuvant Therapy*											
KEYNOTE-564, Choueiri et al.	2024	Pembrolizumab	Placebo	496/498	60 (range: 27-81)/60 (range: 25-84)	158(31.9)/172(34.5)	NA	NA	NA	0.77 (0.60-0.98)/0.67(0.48-0.93)$^\dagger$	
CheckMate 914, Motzer et al.	2023	Nivolumab + Ipilimumab	Placebo	405/411	58 (IQR: 51-65)/57 (IQR: 50-65)	112(28)/110(27)	NA	NA	NA	0.96 (0.71-1.31)/0.88(0.54-1.44)$^\dagger$	
IMmotion010, Pal et al.	2022	Atezolizumab	Placebo	390/388	60.5 (IQR: 52-69)/60 (IQR: 52.8-68)	142(36)/140(36)	NA	NA	NA	0.91 (0.69-1.19)/0.96(0.63-1.36)$^\dagger$	
PROSPER, Allaf et al.	2022	Nivolumab	Placebo	404/415	60 (range: 31-88)/61 (range: 23-88)	133(32.9)/138(33)	NA	NA	NA	0.78 (0.57-1.08)/1.34(0.88-2.05)$^\dagger$	

*RCTs* randomized controlled trials, *ICI* immune checkpoint inhibitors, *RCC* renal cell carcinoma, *OS* overall survival, *PFS* progression-free survival, *DFS* disease-free survival, *HR* hazard ratio, *CI* confidence interval, *NA* not available, *IQR* interquartile range, *IMDC* International Metastatic RCC Database Consortium, *T* treatment, *C* Control

* MSKCC risk classification was used instead of IMDC for CheckMate 025

$^\dagger$ Values represent HR (95% CI) of DFS

### Hospital analysis

#### Cohort characteristics

In our hospital’s study on ccRCC, we compared young and old patient groups using chi-square and univariate Cox regression analyses. Despite the lack of statistically significant differences in most analyses due to a small sample size and limited follow-up period, several notable trends emerged. Gender distribution was comparable between groups, with no significant impact on ccRCC incidence. Tumor characteristics, including grade and TNM classification, showed no significant differences between age groups (P > 0.05). Surgical approaches were consistent across ages, with partial nephrectomies less common in both groups (Table 4).

**Table 4:** Clinico-pathological characteristics of clear cell renal cell carcinoma from hospital cohort.

Parameters		Young(<65 years)n (%)	Old ($\ge$65 years)n (%)	P- value
	All cohort (53)	34 (45.6%)	19 (35.8%)	
Gender	Male	20 (58.8%)	12 (63.2%)	0.757
	Female	14 (41.2%)	7 (36.8%)	
TNM	T1,T2.N0M0	25 (73.5%)	13 (68.4%)	0.69
	Others	9 (26.5%)	6 (31.6%)	
Grade	Low	19 (55.9%)	10 (52.6%)	0.8
	High	15 (44.1%)	9 (47.4%)	
Surgery	Partial	9 (26.5%)	5 (26.3%)	0.9
	nephrectomy			
	Total	25 (73.5%)	14 (73.6%)	
	nephrectomy			
Laterality	Right	13 (38.2%)	8 (42.1%)	0.78
	Left	21 (61.8%)	11 (57.9%)	

Pearson chi square test

#### Immunohistochemistry findings

**Fig. 12:**
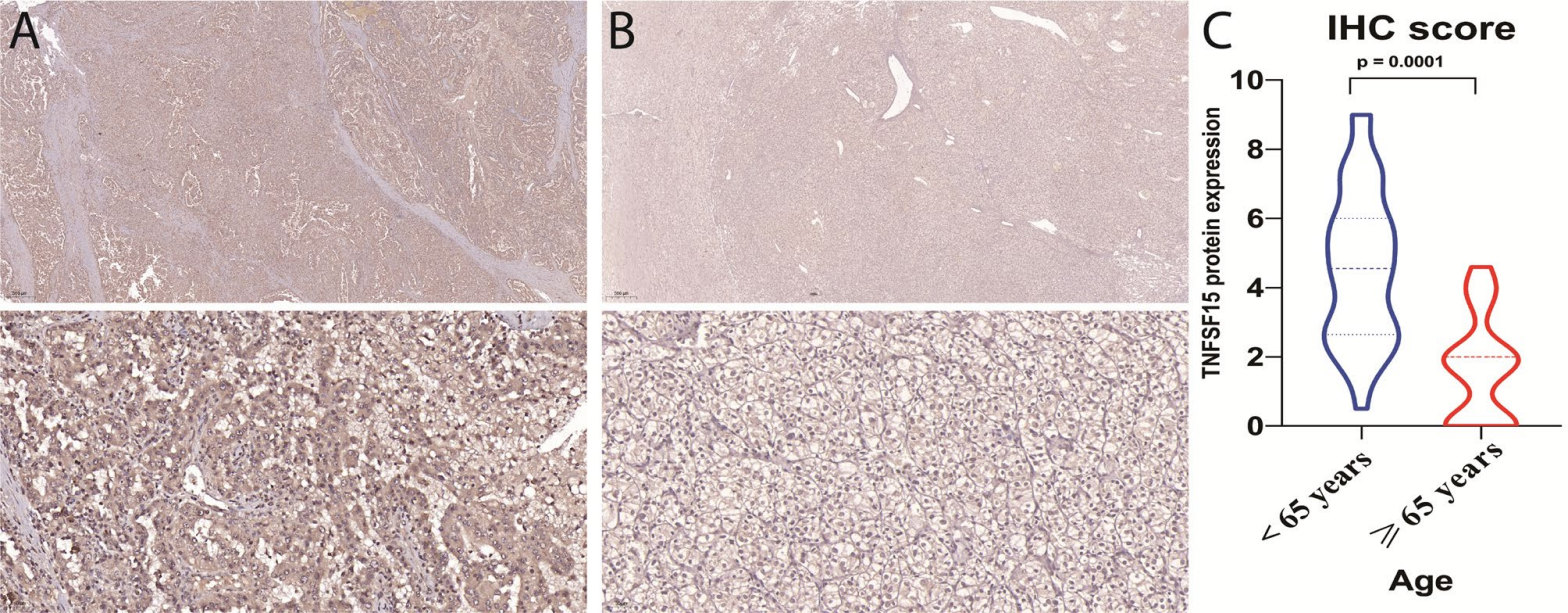
Immunohistochemistry (IHC) analysis of TNFSF15 gene expression differences between younger and older groups in the hospital cohort via IHC. (**A**) and (**B**) display IHC staining results for the younger and older groups, respectively (scale bar: 500 $\mu$m upper panel, 50 $\mu$m lower panel). (**C**) IHC scores quantification between the two groups using the Mann–Whitney U-test, demonstrating significantly higher TNFSF15 expression in younger ccRCC patients (P = 0.0001).

To validate our previously published findings for aging-related immune genes in ccRCC^40^, we performed IHC on ccRCC samples collected from our hospital. Correlation analysis revealed a significant negative relationship between TNFSF15 expression and chronological age (Spearman correlation = -0.6, p < 0.0001) (Fig. S9. Specifically, TNFSF15 expression was significantly higher in younger patients compared to older patients with an IHC score p value equal to 0.004 (Fig. 12). These findings provide robust validation of the age-dependent expression patterns of TNFSF15 in ccRCC, suggesting potential implications for age-related differences in immune responses within the tumor microenvironment (Fig. 12).

## Discussion

RCC incidence generally peaks in the seventh decade, with only 3%–5% of cases diagnosed in individuals under 40. Age significantly impacts aRCC prognosis, as older patients often experience poorer outcomes^41^, potentially due to immunosenescence-a process that diminishes immune function, enabling tumor immune evasion and altering RCC biology^42^. Over the last decade, clinical trials have evaluated ICI’ effectiveness in several solid cancer types, including treatment-resistant malignancies like RCC. Data suggest that ICI, as monotherapy or in combination, improve OS in aRCC patients^43,44^. However, research addressing age-specific effects of ICI in aRCC remains limited. Our study investigates ICI effects across age groups, revealing notably higher survival rates in younger patients with aRCC, while no such age-related survival differences existed before ICI introduction.

The ICI era has marked a significant improvement in survival outcomes for aRCC patients, with longer median survival observed across most variables in both age groups compared to the pre-ICI period. Within the ICI era, younger aRCC patients (<65 years) experience significantly better survival outcomes, with age serving as an independent prognostic factor: younger patients show a lower HR for death than older patients ($\ge$65 years). Prior studies indicate that multimodal therapies based on ICI enhance anticancer efficacy across various illnesses^45^. While some research suggests minimal age-related differences in outcomes with immunotherapy^46–48^, our analyses demonstrate a clear age-associated disparity in the ICI era. Univariate analyses suggest that older individuals tend to have lower life expectancy than younger patients^49^, though healthcare quality in universal healthcare systems may influence these results^50,51^. However, these factors alone do not fully explain the age-related disparities we observed.

Our findings highlight that patients with certain demographic and clinical characteristics-specifically Black race, poor or undifferentiated tumor grades, and tumor sizes >7 cm-face poorer prognoses across age groups. These factors should be considered when stratifying the risk and planning treatment approaches for patients with aRCC in the ICI era. While our SEER analysis identified an association between surgical intervention and improved survival across age groups in the ICI era, this finding must be contextualized within the evolving evidence on cytoreductive nephrectomy (CN). Landmark trials, CARMENA and SURTIME, demonstrated that immediate CN was not superior to systemic therapy alone or deferred CN in metastatic RCC^38,39^. The apparent survival benefit in our analysis likely reflects a selection bias, as better-performing patients typically undergo surgery. Current evidence suggests that CN should be considered selectively for patients with good performance status, intermediate-risk disease, and limited metastatic burden^52–54^, while avoiding CN in IMDC poor-risk patients^55,56^. Evidence increasingly favors deferred CN after initial systemic therapy, with SURTIME showing a significant OS advantage for this approach (32.4 vs. 15.0 months)^39^. Several ongoing trials have evaluated CN specifically with immune checkpoint inhibitors^57–60^.

Prognostic analyses using nomograms and ROC testing revealed that metastatic status, in addition to surgery, grade, and tumor size, critically influences OS, with metastatic patients exhibiting lower OS than non-metastatic cases. Liver and brain metastases correlate strongly with reduced survival, especially in older patients who generally have worse outcomes^61,62^.

Our meta-analysis of major RCTs in aRCC demonstrated that ICI-based therapies significantly improved OS in patients aged <65 years compared to those $\ge$65 years, with a striking 29% reduction in mortality risk (pooled OR = 0.71; 95% CI 0.61–0.83, p < 0.0001). Importantly, conventional non-ICI therapies showed no significant age-dependent treatment effects (OR, 0.88; 95% CI 0.75–1.02; p = 0.10). This distinct pattern suggests that age-related factors specifically influence responses to immunotherapy. While immunosenescence, characterized by age-related declines in T cell activation and function, likely plays a central role^35,36,40^, several other factors may contribute to the observed survival disparities. These include altered pharmacokinetics owing to age-related changes in drug metabolism, higher treatment discontinuation rates driven by toxicity or patient preference, and the presence of comorbidities requiring concomitant medications^63,64^. Although a recent meta-analysis of various solid cancers showed no age-related survival differences, specific RCC trials (KEYNOTE-426, JAVELIN Renal 101, and CheckMate 9ER) indicate that chronological age is a predictive factor for aRCC patients treated with ICI^37,65^. Although other clinical and demographic factors may influence ICI effectiveness, age remains a significant prognostic factor for aRCC^24,65^. Our secondary outcome data on PFS showed benefits favoring the younger group compared with non-ICI treatments, although these did not reach statistical significance. The value of PFS as a surrogate endpoint for ICI in aRCC remains debated^13,20,66–68^, as the traditional RECIST criteria may not fully capture immune therapy responses. The iRECIST framework, which incorporates immune-related changes, could offer more precise prognoses for ICI therapy, although further validation in RCC is necessary^67,69^.

IHC results from our hospital cohort confirmed that TNFSF15, a previously identified aging-related gene, showed significantly higher expression in younger patients^35,40^. These findings support the immunosenescence hypothesis but do not exclude the additional contributing factors mentioned above. TNFSF15 could serve as a valuable prognostic biomarker, particularly for predicting survival outcomes in the ICI era and for enhancing clinical decision-making for age-based treatment strategies.

Treatment decisions must consider the distinct toxicity profiles of ICI, particularly in the older population. Limited data indicate that older patients may have a higher risk of immune-related AEs^61,62,65^, with evidence showing that they are more likely to develop multisystem irAEs (32% vs. 18%) and require hospitalization (54% vs. 29%) than their younger counterparts^70,71^. ICI discontinuation rates increase substantially in the very elderly population (30.9% in 90+ years vs. 15.1% in 80-89 years)^72^. Thyroid endocrinopathies often present atypically in older adults as fatigue or cognitive changes^73^, whereas musculoskeletal toxicities may disproportionately affect function in those with baseline mobility limitations^74,75^. Management is further complicated by comorbidities and polypharmacy, with corticosteroids carrying substantial risks including infections, delirium, and worsening sarcopenia^76^.

This study had several limitations. The SEER database lacks age-specific data for aRCC with limited OS follow-up (24 months) and no insight into comorbidities or performance status. Critically, SEER cannot distinguish between curative nephrectomy and CN, making the interpretation of surgical survival benefits challenging. As with all retrospective studies and meta-analyses, this study is subject to inherent biases and a lack of patient-level stratification. Another limitation is the lack of external validation for our prognostic nomograms, which requires testing across independent datasets to ensure generalizability. Our meta-analysis included only RCTs with variations in demographics and therapies, making age comparisons difficult^9,77^, especially because older patients comprised only 37% of the sample. Given these limitations, more extensive, balanced studies focusing on age-specific clinicopathological factors are needed to optimize both ICI treatment and surgical intervention strategies for different age groups in aRCC. Future research should aim to disentangle the complex interplay between immunosenescence, pharmacokinetics, and comorbidities in determining treatment outcomes in older adults with advanced renal cell carcinoma.

## Conclusion

In summary, our findings indicate that age-related differences in the ICI era significantly impact OS outcomes for aRCC patients, with younger patients (<65 years) experiencing a more substantial survival benefit from ICI-based therapies than older patients ($\ge$65 years). Although PFS outcomes varied between age groups, these differences did not reach statistical significance. Our analysis identified an association between surgical intervention and improved survival in the ICI era, but this finding should be interpreted within the context of evolving evidence about cytoreductive nephrectomy. Furthermore, grade, tumor size, and distant metastasis to major organs (bone, brain, liver, and lung) are essential prognostic factors, with considerable disparities observed in prognosis, particularly during the ICI period. The elevated expression of the age-related immune marker TNFSF15 in younger patients may further elucidate these age-specific responses. These findings provide essential insights for clinical decision-making, particularly for evaluating the risks and benefits of ICI therapy in old aRCC patients with aRCC. To deepen our understanding of the effects of ICI therapy in older populations, further studies with larger cohorts are warranted.

## Materials and methods

### SEER database

#### Data sources, ethics, and patient cohort

Authorization to access the Surveillance, Epidemiology, and End Results (SEER) database was obtained following registration and compliance with the database’s policies. We accessed the SEER database on September 20, 2024, utilizing the SEER Research Data, November 2023 release, which covers the years 2000 to 2021. Data extraction and analysis were conducted using SEER*Stat software (version 8.4.4). As per SEER program policies, informed consent from individual patients was not required for this analysis.

We identified advanced RCC cases (stages III and IV) using the International Classification of Diseases for Oncology, Third Edition/World Health Organization 2008 (ICD-O-3/WHO 2008) classification system. Staging was aligned with the eighth edition of the American Joint Committee on Cancer (AJCC) staging system, which was adapted to match the historical data from the sixth edition (2004–2015) and the Derived SEER Combined Staging Group (2016 onwards) for consistency. Our cohort included microscopically confirmed cases under active follow-up, focusing on the first primary or first-only cases.

Overall survival (OS) was calculated from the date of advanced RCC diagnosis, with a maximum follow-up duration of 24 months, to ensure a balanced comparison across treatment periods. We defined two distinct periods, 2004–2015 and 2016–2021, with the latter period marked by the increasing clinical adoption of immune checkpoint inhibitors (ICIs) following the FDA approval of nivolumab in November 2015^9,10^. It is important to note that this temporal division serves as a proxy for potential ICI availability rather than confirming the actual ICI treatment, as the SEER database does not provide specific treatment information beyond surgery and radiation therapy. Patients were categorized into younger (<65 years), intermediate (65-74 years), and older ($\ge$75 years) age groups to allow for more granular age-based analysis.

The dataset included comprehensive patient information, including demographic characteristics (age, sex, and race), clinical parameters (tumor size categorized as <4 cm, 4–7 cm, and >7 cm; grade classified as well/moderate versus poor/undifferentiated; metastatic sites to bone, brain, liver, and lung available for 2011–2021), treatment details (surgery, radiation, and chemotherapy status), and socioeconomic factors (marital status and income level categorized as < $60,000 vs. > $60,000). Fig. 1 presents a detailed study flow diagram with the inclusion and exclusion criteria.

#### SEER statistics

The essential characteristics of the patients with aRCC were compared using the chi-square test. Overall survival was analyzed using Kaplan-Meier curves with log-rank tests to calculate the median survival months from diagnosis. Both univariate and multivariate analyses were performed using Cox proportional hazards models to identify the significant prognostic factors. All survival analyses explicitly refer to overall survival (OS) unless otherwise specified. We acknowledge that the SEER database lacks information on performance status, comorbidities, and specific systemic therapies, which is an inherent limitation of this analysis.

Based on the significant parameters identified in the multivariate Cox regression model, we constructed a nomogram and developed a risk-categorization system. The predictive accuracy of the nomogram for estimating mortality risk across age groups was evaluated using receiver operating characteristic (ROC) curves. All statistical analyses were conducted using R version 4.2.2 and SPSS 26, with statistical significance defined as $p<0.05$ and a critical value set at $p<0.0001$.

### Meta-analysis

#### Methods

The study protocol was submitted to the International Prospective Register of Systematic Reviews (PROSPERO: CRD42024595559) for official registration.

#### Searching approach

This systematic review and meta-analysis was conducted in accordance with the recommendations established by the Preferred Reporting Items for Systematic Reviews and Meta-Analyses (PRISMA) for observational studies in epidemiology (Table S3)^78^.

To locate studies that evaluated the oncologic outcomes of ICI-based therapies in advanced RCC across different age groups, a comprehensive search was carried out in September 2024 using PubMed, Web of Science, and Scopus databases. No limits were imposed on publication date or language. The search terms included variants of “metastatic” or “advanced,” “renal cell carcinoma” or “renal cancer” or “kidney cancer,” “randomized” or “randomly,” and “immunotherapy” or “immune checkpoint inhibitor.”

The specific Scopus terms used were as follows: 1. TS=(immunotherapy) OR TS=(immune checkpoint inhibitor) OR TS=(nivolumab) OR TS=(ipilimumab) OR TS=(pembrolizumab) OR TS=(tremelimumab), 2. TS=(metastatic) OR TS=(advanced); 3. TS=(randomized) OR TS=(random), with the search combining queries 1, 2, and 3. This yielded 5,776 articles from Scopus, 845 articles from the Web of Science using a similar search string, and 425 articles from PubMed, totaling 7,046 articles. After removing duplicates, 6,015 articles remained in the initial search of 8,522 records. Subsequently, 6,418 articles were screened based on their titles and abstracts. Of these, 50 articles were selected for full-text review, which ultimately identified ten randomized controlled trials^13–20,79–81^. These trials included a total cohort of 8,434 patients, of whom 4,207 received immune checkpoint inhibitor (ICI) therapy and 4,227 received other treatment modalities or placebo (Fig. 7).

#### Study outcomes

The primary outcome of interest was overall survival (OS), defined as the time from randomization to death from any cause, as reported in five studies. Secondary outcomes included progression-free survival (PFS) in five studies, defined as the period from randomization to disease progression or death; disease-free survival (DFS) in three adjuvant trials, defined as the time from randomization to disease recurrence or death; and objective response rate (ORR) data available for four studies, defined as the percentage of patients achieving complete or partial response according to RECIST v1.1.

All survival outcomes were reported as hazard ratios (HRs) with 95% confidence intervals (CIs), whereas ORR was reported as odds ratios (ORs) with 95% CIs. Each outcome was clearly labeled in all analyses and figures to maintain transparency and avoid confusion.

#### Inclusion, exclusion criteria, and data extraction

**Inclusion and exclusion criteria** Studies were included if they investigated patients with RCC (patients) with either metastatic disease or high-risk localized disease requiring adjuvant therapy and compared the efficacy of ICI monotherapy or ICI-based combination therapy (interventions) with the efficacy of standard of care (SOC) at the time of study enrollment (comparisons) to assess their differential effects on OS, PFS, or DFS between age groups (Outcome) in an RCT (Study design). For metastatic RCC, studies reporting OS and PFS were included in our primary analysis, whereas for localized RCC in the adjuvant setting, studies reporting DFS were included.

Studies lacking original patient data, reviews, letters, editorial comments, replies from authors, case reports, or articles not written in English were excluded. The references of all included papers were scanned for additional relevant studies.

**Data extraction** Two authors independently extracted data from each study and a third investigator resolved any discrepancies. The following data were extracted: first author’s name, publication year, inclusion criteria, treatment agents, median patient age, stratification of patients by age subgroups (<65 years and $\ge$65 years), follow-up duration (median and range), International Metastatic RCC Database Consortium (IMDC) risk classification (when available), performance status distribution, and number of events in each age group.

We specifically separated the data from metastatic RCC trials and adjuvant therapy trials to ensure an appropriate clinical context for interpretation. Only patients receiving ICI-based therapy were included in the ICI group, whereas those in the control arms were allocated to the non-ICI group. We acknowledge that RCTs typically include patients with good performance status and limited comorbidities, which may not fully represent the general population of older patients with RCC.

#### Risk of bias assessment

The quality of the included studies and risk of bias were assessed using the risk-of-bias instrument (RoB version 2) included in the Cochrane Handbook for Systematic Reviews of Interventions (Fig. 10)^82^. Two reviewers independently evaluated each study across the domains of the randomization process, deviations from intended interventions, missing outcome data, measurement of outcomes, and selective reporting. Any discrepancies in the evaluations were resolved through discussion with a third reviewer.

#### Meta-analysis statistics

Forest plots were constructed for the meta-analysis to investigate the association between ICI treatment and oncological outcomes across age groups. We primarily used odds ratios (ORs) for the main analysis, with supplementary analyses using hazard ratios (HRs) when reported in the original studies, allowing for a more comprehensive assessment of time-to-event outcomes.

The calculation of effect estimates employed either fixed- or random-effects models based on heterogeneity assessment^83^. Cochran’s Q test and the I$^{2}$ statistic were used to quantify the heterogeneity. When the heterogeneity was low (p > 0.05, I$^{2}<$ 50%), a fixed-effects model was applied. For significant heterogeneity (p <0.05, I$^{2}\ge$ 50%), a random-effects model was used to account for between-study variation^84^.

Studies were stratified by clinical setting (metastatic first-line therapy, metastatic second/third-line therapy, and adjuvant therapy) and treatment group (ICI vs. non-ICI) to ensure appropriate contextual interpretation. We conducted predefined subgroup analyses based on the ICI type (anti-PD-1/PD-L1 alone vs. ICI combinations), patient performance status (when available), sex distribution, and follow-up duration.

Sensitivity analyses were performed by sequentially excluding each study to assess its influence on the pooled effect estimate. An additional sensitivity analysis, excluding studies with a high risk of bias in any domain, was conducted to ensure the robustness of the findings.

All analyses were performed using Review Manager 5.4 (RevMan, Cochrane Collaboration, Oxford, UK). Throughout this study, a significance level of p < 0.05 was applied for statistical testing.

### Hospital data

#### Patient cohort

Human clear cell renal cell carcinoma (ccRCC) tissues were obtained from 53 patients in different age groups undergoing surgical resection at the First Affiliated Hospital of Dalian Medical University (Dalian, China) between January 2016 and December 2021. Two pathologists confirmed the tumor grades of these tissues. All patients provided written informed consent, and the Medical Ethics Committee of the First Affiliated Hospital of Dalian Medical University (PJ-KS-KY-2023-353) approved the study. All methods were performed in accordance with the relevant guidelines and regulations, including the Declaration of Helsinki and institutional ethical standards.

#### Immunohistochemistry (IHC) analysis

Tissue samples were fixed overnight in 4% paraformaldehyde to obtain paraffin-embedded sections. The sections were deparaffinized with xylene and rehydrated using an alcohol gradient. After repairing the antigen with Tris-EDTA buffer(pH9.0), endogenous peroxidase was removed by treating slides with an endogenous catalase blocker (3%). The tissues were then incubated with a TNFSF15 Rabbit Polyclonal Antibody (1:200 dilution, HUABIO, Catalog ER1917-90) overnight at 4$^{\circ }$C. The next day, it was incubated with enzyme-labelled goat anti-rabbit IgG polymer, followed by treatment with diaminobenzidine (DAB). Finally, the sections were stained with hematoxylin and dehydrated using an alcohol gradient. The slides were finally mounted using cover slips on the tissue using Neutral Balsam (Solarbio Life Sciences, Cat# G8590) and allowed to dry. The slides were scanned using a KF-PRO-005 digital pathology scanner(KFBIO, Ningbo City, China). The staining intensity was scored according to the number of chromogenic cells: the number of positive cells <1/3 was 1 point, the number of positive cells 1/3-2/3 was 2 points, the number of positive cells $\ge$2/3 was 3 points, and according to the color depth of cells, the score of cells without positive reaction was 0, light yellow was 1, brown-yellow was 2, and tan was 3. The final scores for each slide were calculated by multiplying the scores for intensity and percentage (A$\times$B=0 is (-), A$\times$B=1   2 is (+), A$\times$B=3   4 is (++), A$\times$B=6   9 is (+++).

## Supplementary Information

Supplementary Information 1.

Supplementary Information 2.

## Data Availability

The datasets generated and analyzed during this study are available in the SEER database at [https://seer.cancer.gov/data/]. The article and its Supplementary Material include all original contributions presented in this study. For additional information, don’t hesitate to get in touch with the corresponding author.
