# Supplementary material for: Age-Related Survival Disparities in Advanced Renal Carcinoma in the Immune Checkpoint Inhibitor Era using the SEER Database and Meta-analysis
Source: Sci Rep. 2025 Dec 3;15:43501. doi: 10.1038/s41598-025-06297-y (PMC12695934; doi:10.1038/s41598-025-06297-y)

**Additional Information**

**Supplementary Information:**

**Supplementary Tables**

• Table S1. Univariate and multivariate analysis of factors affecting survival in the non-ICI and ICI eras in the young age group of advanced renal cell carcinoma patients.

• Table S2. Univariate and multivariate analysis of factors affecting survival in the non-ICI and ICI eras in the old age group of advanced renal cell carcinoma patients.

• Table S3. PRISMA checklist 2009 for meta-analysis for ccRCC RCTs

**Supplementary Figures**

• S1 Fig. Kaplan-Meier survival curves comparing overall survival between non-ICI and ICI eras. (A) Survival curve for the young age group (*<* 65 years). (B) Survival curve for the old age group (*≥* 65 years).

• S2A Fig. Survival analysis comparing patients with and without metastases during the ICI era, displayed by age group: Left panels represent patients younger than 65 years (*<* 65 years), and right panels represent patients 65 years and older (*≥* 65 years). Upper row: Bone metastases (Yes vs. No). Lower row: Brain metastases (Yes vs. No).

• S2B Fig. Survival analysis comparing patients with and without metastases during the ICI era, displayed by age group: Left panels represent patients younger than 65 years (*<* 65 years), and right panels represent patients 65 years and older (*≥* 65 years). Upper row: Liver metastases (Yes vs. No). Lower row: Lung metastases (Yes vs. No).

• S3 Fig. Survival analysis comparing patients with metastases in different organs (bone, brain, liver, and lung) during the ICI era. Left panels represent the younger age group (*<* 65 years), and right panels represent the older age group (*≥* 65 years).

• S4 Fig. Survival analysis across three age groups (*<* 65 years, 65*−*74 years, and *≥* 75 years) in the ICI era. (A) Kaplan–Meier survival curves for the three age groups. (B) Multivariate Cox regression model in the ICI era for the three age groups. (C) Cox survival curves illustrating cumulative survival for each age group in the ICI era, with HR for comparison between age groups (*<* 65 years vs. 65*−*74 years

and *≥* 75 years).

• S5 Fig.Funnel plots assessing publication bias in the meta-analysis. (A) ICI treatment subgroups showing distribution of studies across adjuvant ICI monotherapy, first-line ICI combination therapy, and second/third-line ICI monotherapy. Studies are relatively symmetrical around the vertical axis, suggesting minimal publication bias. (B) Control treatment subgroups displaying distribution across first-line, second/third-line, and adjuvant therapy arms. The balanced scatter pattern indicates low likelihood of publication bias in both treatment groups.

• S6 Fig. Meta-analysis of immune checkpoint inhibitor (ICI) therapy secondary outcomes comparing younger (*<* 65 years) versus older (*≥* 65 years) advanced renal cell carcinoma (aRCC) patients. (A) Disease progression analysis shows no significant difference between age groups (OR = 0.85, 95% CI: 0.59-1.22, p = 0.37), with substantial heterogeneity (I² = 74%). (B) Objective response rates reveal comparable outcomes in first-line ICI combination therapy (OR = 1.02, 95% CI: 0.68-1.53, p = 0.91) and second/third-line monotherapy (OR = 0.74, 95% CI: 0.47-1.16, p = 0.19), with overall pooled OR = 0.96 (95% CI: 0.68-1.36, p = 0.83). (C) Recurrence analysis indicates a trend favoring younger patients (OR = 0.84, 95% CI: 0.67-1.04, p = 0.10) with no significant heterogeneity (I² = 0%).

• S7 Fig. Meta-analysis of control treatment secondary outcomes in advanced renal cell carcinoma (aRCC) comparing younger (*<* 65 years) versus older (*≥* 65 years) patients. (A) Disease progression analysis shows significantly higher risk in older patients (OR = 1.42, 95% CI: 1.18-1.71, p = 0.0002) with moderate heterogeneity (I² = 48%). (B) Objective response rates show no significant age-related differences in first-line (OR = 1.03, 95% CI: 0.82-1.28) or second/third-line therapy (OR = 0.85, 95% CI: 0.36-2.01), with overall OR = 1.01 (95% CI: 0.82-1.25, p = 0.90). (C) Recurrence analysis demonstrates a trend favoring younger patients (OR = 0.84, 95% CI: 0.68-1.03, p = 0.10) without significant heterogeneity.

• S8 Fig. Funnel Plots Assessing Publication Bias in the Meta-Analysis of ICI A-C and Control Treatment Subgroups D-F. ICI treatment subgroups A. PFS, B. ORR, C.DFS; and Control treatment subgroups D. PFS, E. ORR, F.DFS.

• S9 Fig. TNFSF15 gene expression correlation with chronological age. The scatter plot shows the relationship between age (x-axis) and TNFSF15 gene expression levels (y-axis) in the hospital ccRCC subjects. The Spearman correlation coefficient of -0.6 indicates a moderate to strong negative correlation, meaning TNFSF15 expression tends to decrease with advancing age.


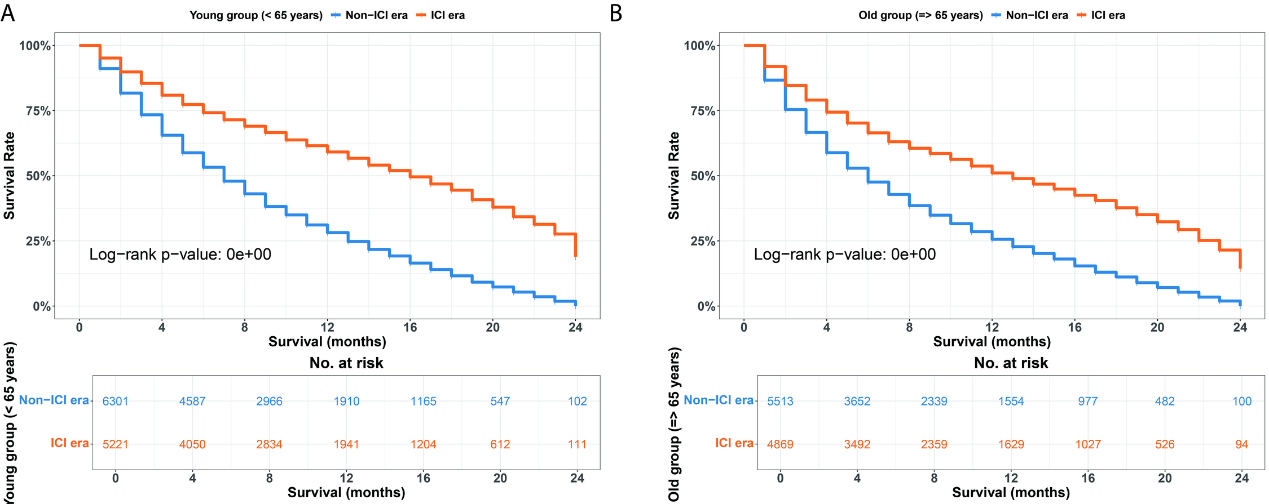


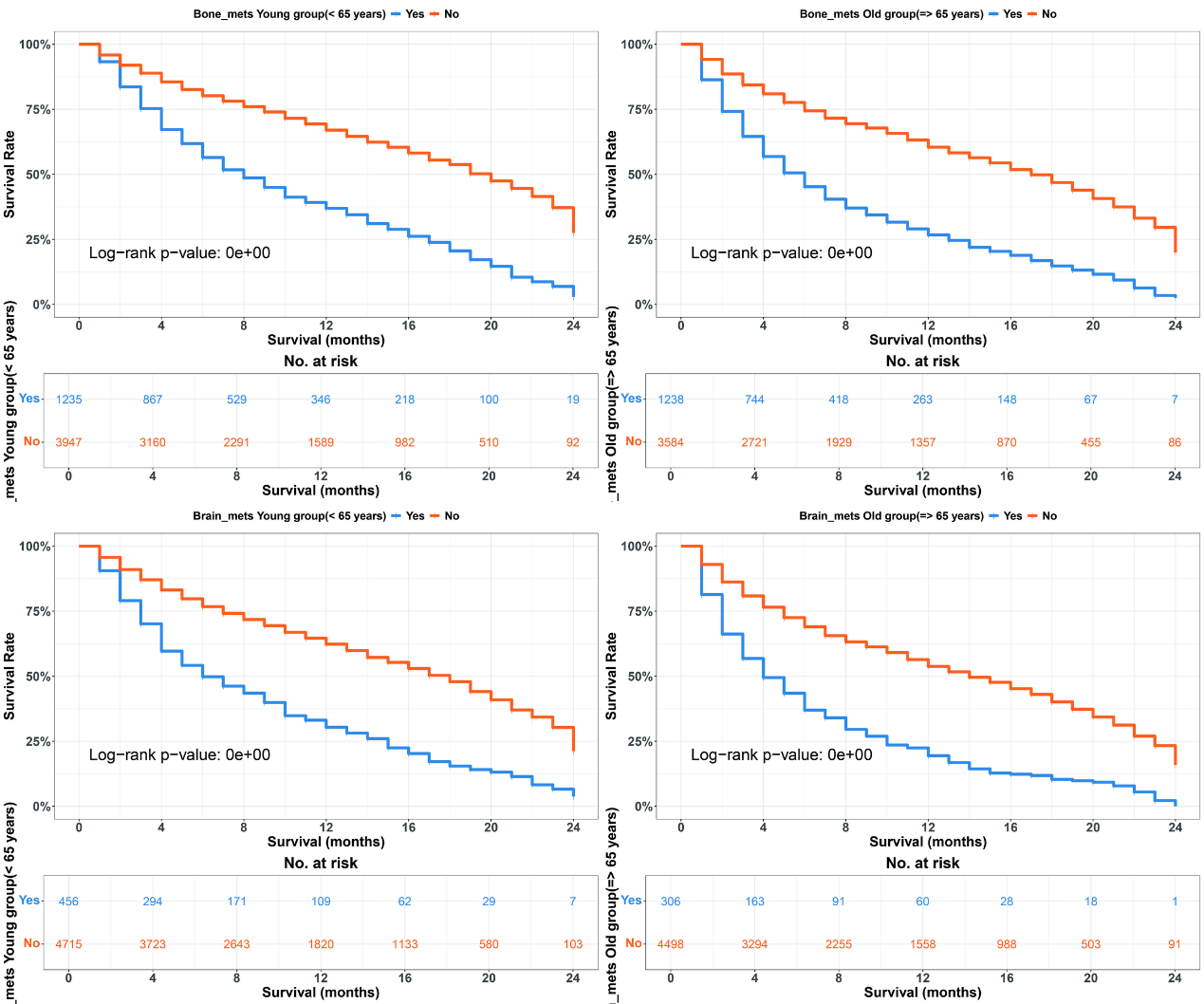


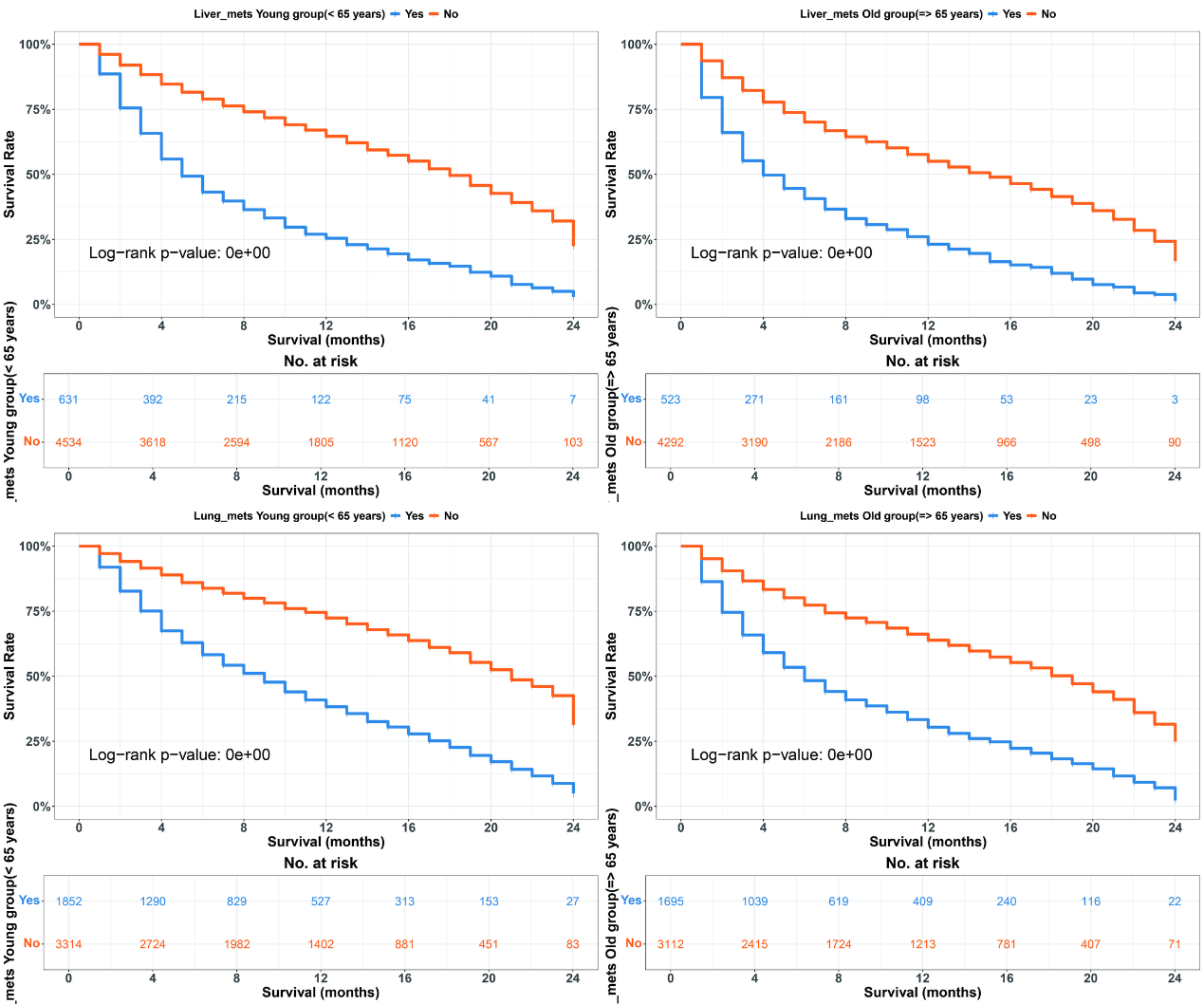


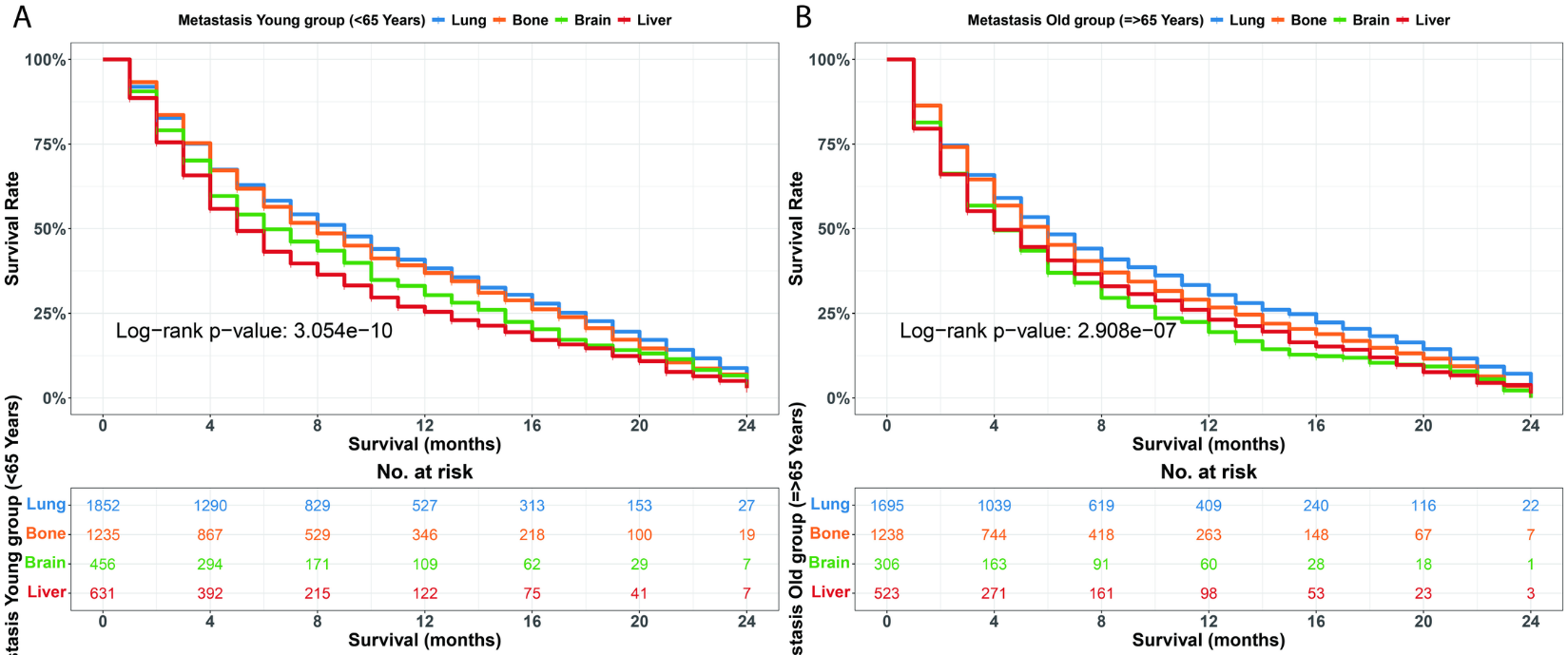

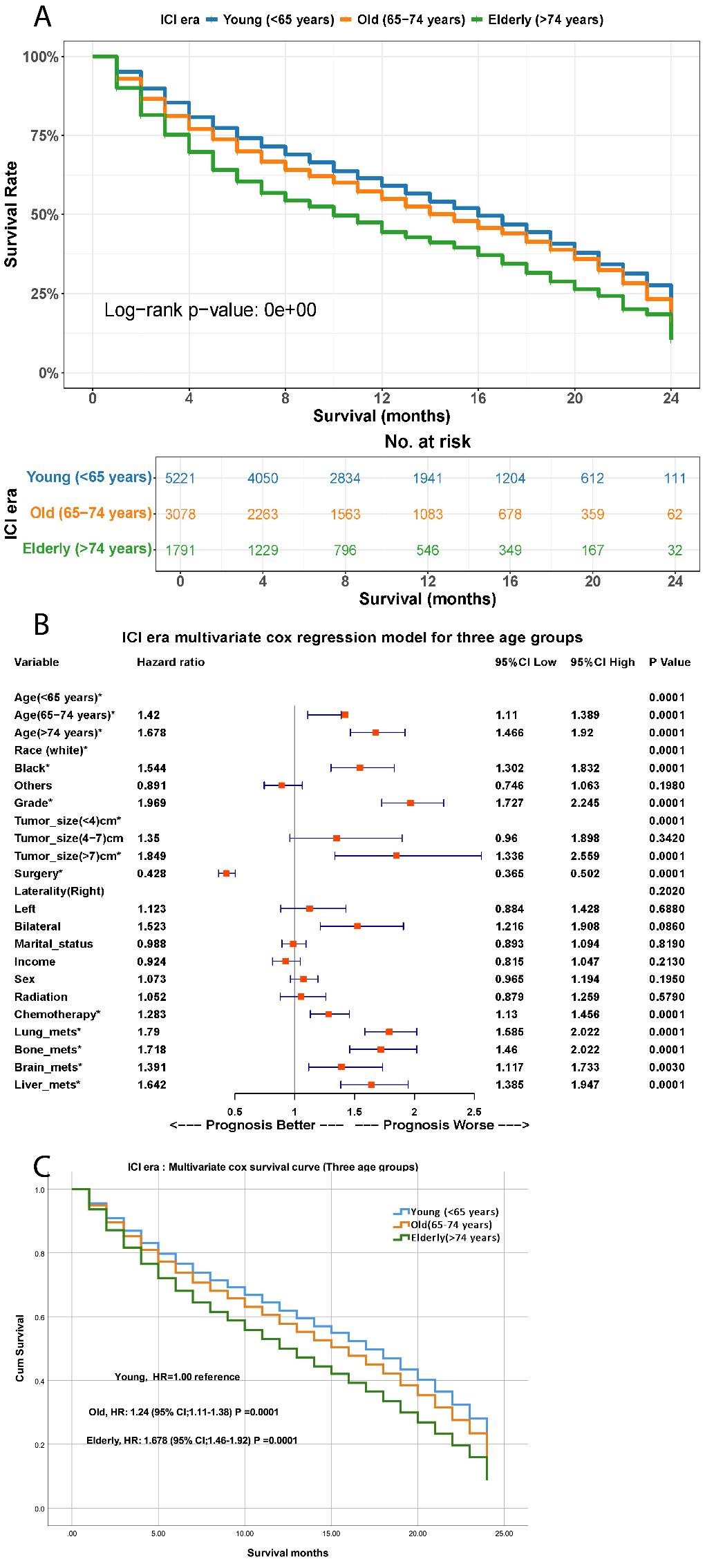





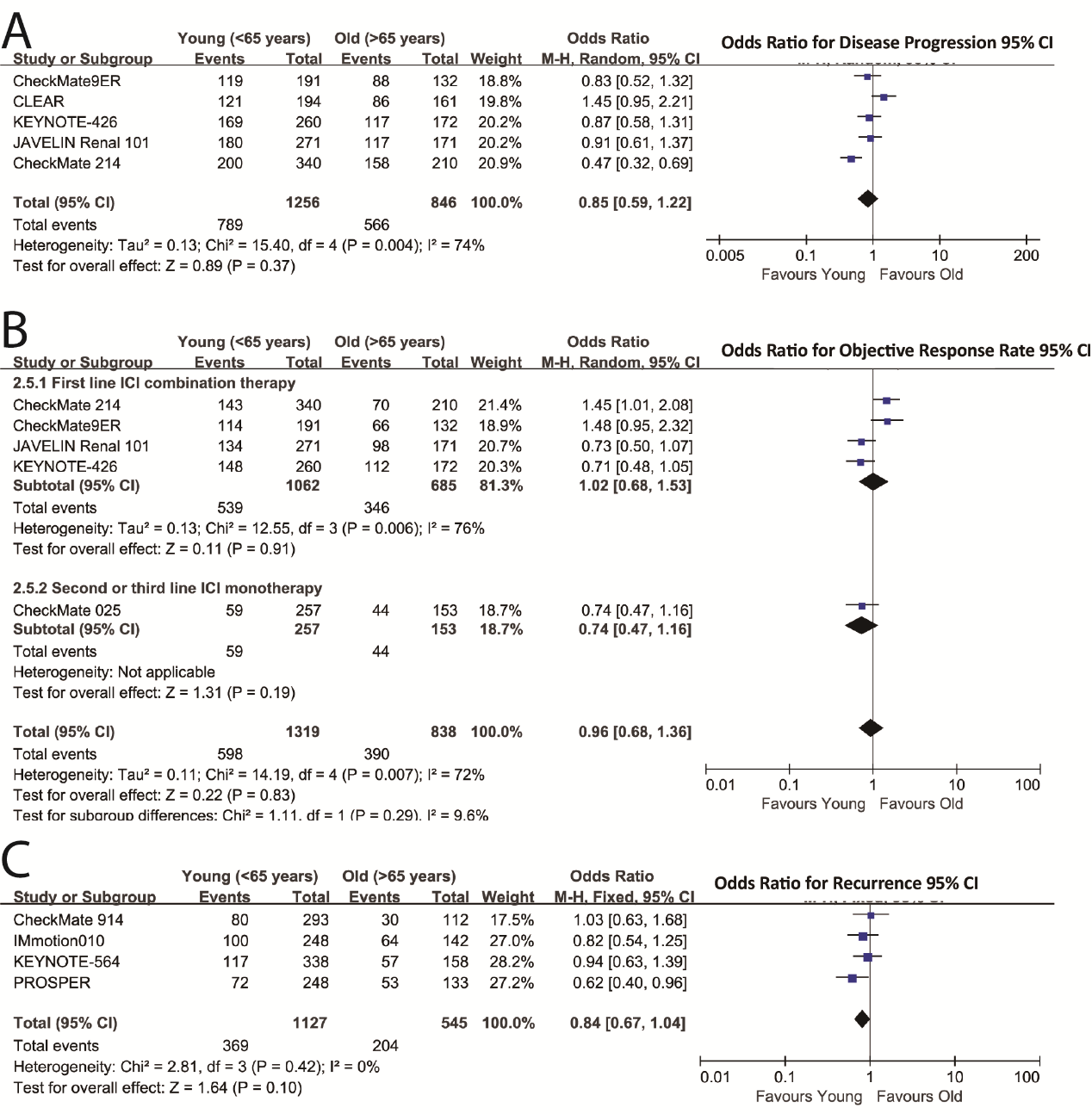


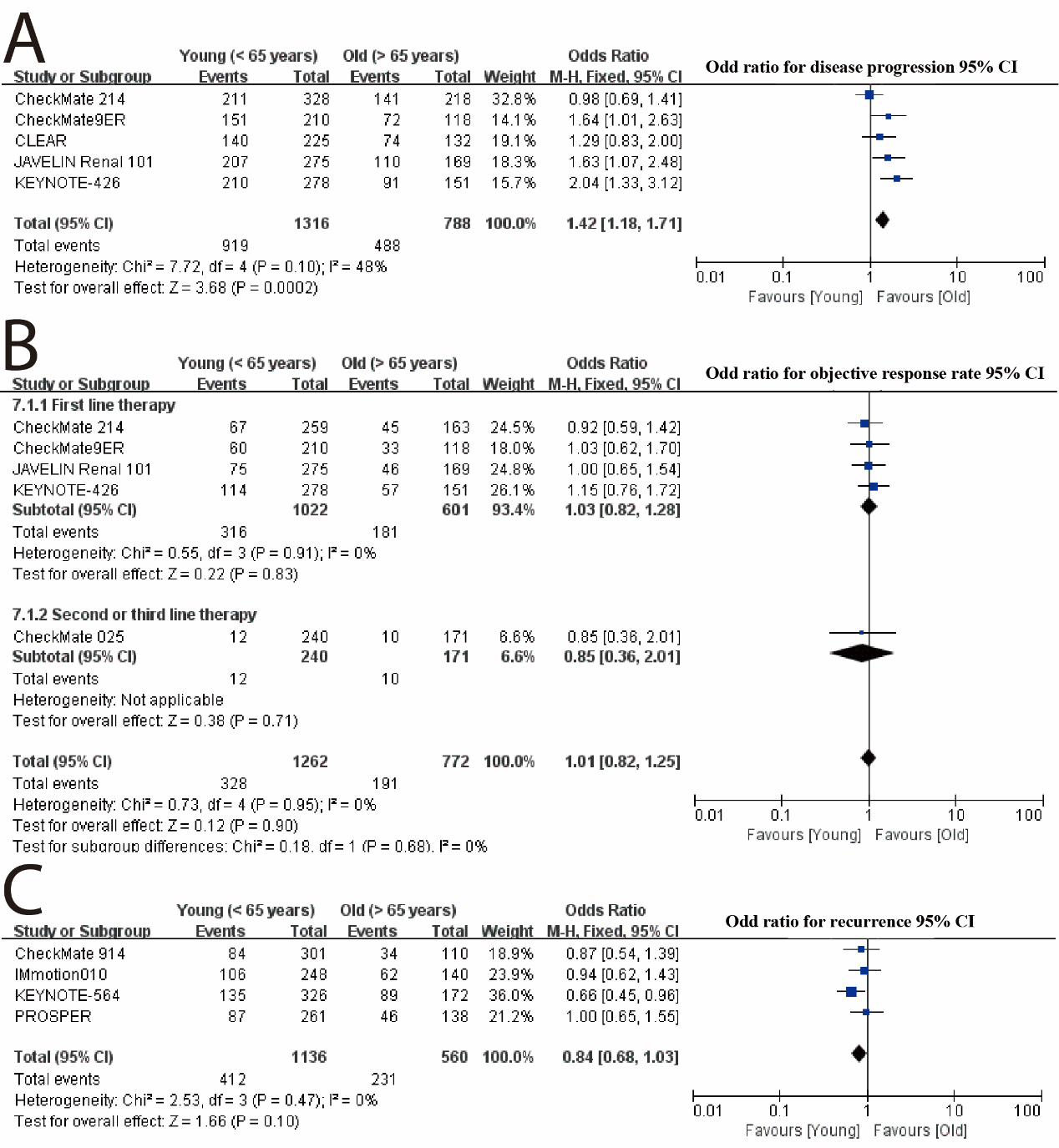


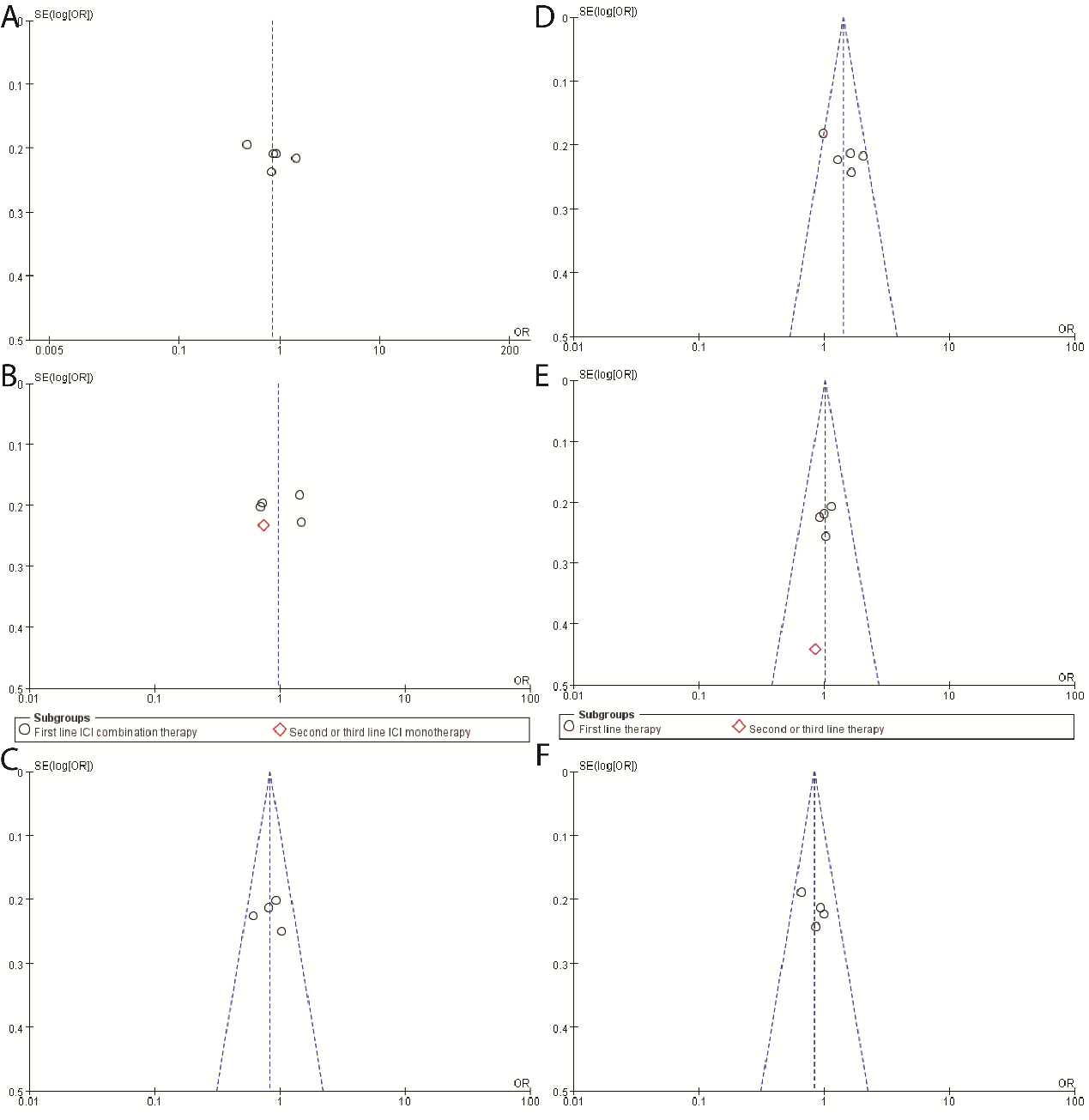


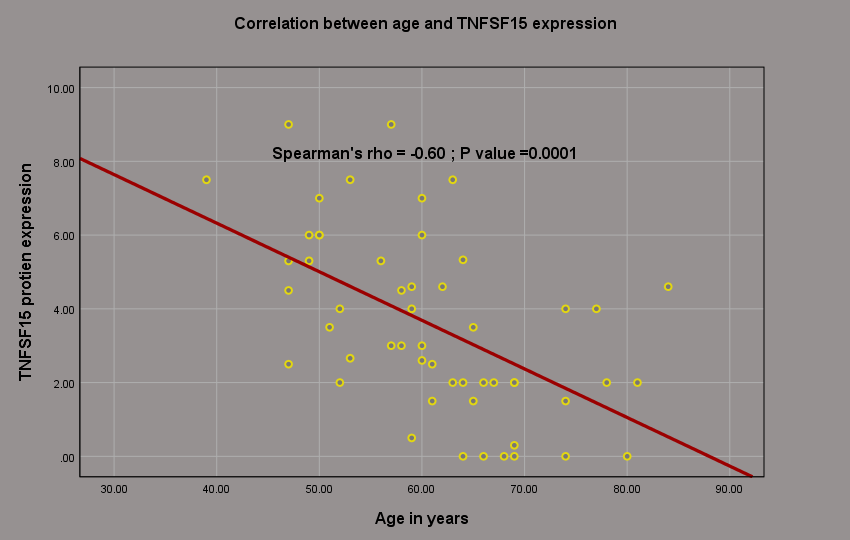

Supplement: Supplementary file 1 — Supplementary Information 1. [file 41598_2025_6297_MOESM1_ESM.docx]
