# Supplementary material for: Age-Related Survival Disparities in Advanced Renal Carcinoma in the Immune Checkpoint Inhibitor Era using the SEER Database and Meta-analysis
Source: Sci Rep. 2025 Dec 3;15:43501. doi: 10.1038/s41598-025-06297-y (PMC12695934; doi:10.1038/s41598-025-06297-y)
Supplement: Supplementary file 2 — Supplementary Information 2. [file 41598_2025_6297_MOESM2_ESM.pdf]

Table 1: **U**nivariate and multivariate analysis of factors affecting survival in the non-ICI and ICI eras in the young group (<65 years) of advanced renal cell carcinoma patients.

3*Parameters	Young group (<65 years) Non-ICI era				Young group (<65 years) ICI era			
	Univariate		Multivariate		Univariate		Multivariate	
	HR (95% CI)	*P* value	HR (95% CI)	*P* value	HR (95% CI)	*P* value	HR (95% CI)	*P* value
**Sex**								
Male vs. female	1.021 (0.966-1.079)	0.468	–	–	1.023 (0.936-1.119)	0.611	–	–
**Race**								
White	Reference	0.002	Reference	0.199	Reference	0.000	Reference	0.0001
Black	1.124 (1.043-1.211)	0.002	1.091 (0.932-1.277)	0.280	1.461 (1.292-1.651)	0.000	1.591 (1.264-2.001)	0.000
Other	0.927 (0.842-1.021)	0.126	0.886 (0.739-1.061)	0.188	0.889 (0.772-1.024)	0.103	0.918 (0.712-1.184)	0.511
**Marital status**								
Yes vs others	1.068 (1.015-1.123)	0.011	0.981 (0.885-1.087)	0.709	1.293 (1.193-1.400)	0.000	1.085 (0.941-1.251)	0.264
**Laterality**								
Right side	Reference	0.002	Reference	0.664	Reference	0.0001	Reference	0.436
Left side	1.030 (0.979-1.084)	0.259	1.022 (0.925-1.130)	0.664	1.089 (1.003-1.181)	0.042	0.947 (0.824-1.089)	0.448
Both side	1.257 (1.105-1.429)	0.001	1.255 (0.766-2.057)	0.368	2.469 (2.011-3.032)	0.000	2.041 (0.504-8.258)	0.317
**Surgery**								
Performed vs. no	1.698 (1.614-1.786)	0.000	1.559 (1.359-1.789)	0.000	3.941 (3.624-4.287)	0.000	2.133 (1.690-2.693)	0.000
**Radiotherapy**								
Yes vs. no	0.889 (0.842-0.938)	0.000	0.982 (0.850-1.136)	0.811	0.500 (0.459-0.546)	0.000	1.143 (0.891-1.467)	0.294
**Chemotherapy**								
Yes vs. no	1.064 (1.012-1.119)	0.015	1.151 (1.026-1.292)	0.016	0.517 (0.477-0.560)	0.000	0.641 (0.542-0.759)	0.000
**Metastasis**								
Bone mets Yes vs. no	0.792 (0.736-0.853)	0.000	0.892 (0.780-1.019)	0.093	0.404 (0.372-0.439)	0.000	0.551 (0.438-0.693)	0.000
Brain mets Yes vs. no	0.649 (0.585-0.721)	0.000	0.784 (0.649-0.947)	0.012	0.418 (0.373-0.469)	0.000	0.679 (0.506-0.912)	0.010
Liver mets Yes vs. no	0.631 (0.576-0.692)	0.000	0.742 (0.637-0.865)	0.000	0.343 (0.311-0.379)	0.000	0.622 (0.493-0.784)	0.000
Lung mets Yes vs. no	0.711 (0.662-0.764)	0.000	0.791 (0.706-0.886)	0.000	0.357 (0.330-0.388)	0.000	0.577 (0.487-0.683)	0.000
**Grade**								
well, moderate vs poor, undifferentiated	1.165 (1.064-1.275)	0.001	1.403 (1.220-1.614)	0.000	2.973 (2.434-3.631)	0.000	2.270 (1.842-2.799)	0.000
**Income**								
<60,000 vs >60,000	0.984 (0.926-1.045)	0.590	–	–	0.902 (0.811-1.002)	0.055	0.862 (0.722-1.029)	0.100
**Tumor size**								
< 4 cm	Reference	0.013	Reference	0.311	Reference	0.001	Reference	0.004
4-7 cm	0.902 (0.798-1.019)	0.098	1.087 (0.835-1.416)	0.534	1.025 (0.853-1.231)	0.792	0.891 (0.633-1.254)	0.508
> 7 cm	0.999 (0.896-1.115)	0.991	1.167 (0.922-1.477)	0.200	1.549 (1.315-1.824)	0.000	1.223 (0.893-1.675)	0.209

Abbreviations: HR: Hazard Ratio; CI: Confidence Interval; ICIs: Immune Checkpoint Inhibitors

Table 2: **U**nivariate and multivariate analysis of factors affecting survival in the non-ICI and ICI eras in the Old group (≥65 years) of advanced renal cell carcinoma patients.

3*Parameters	Old group (≥65 years) Non-ICI era				Old group (≥65 years) ICI era			
	Univariate		Multivariate		Univariate		Multivariate	
P value	HR (95% CI)	*P* value	HR (95% CI)	*P* value	HR (95% CI)	*P* value	HR (95% CI)	
**Sex**								
Male vs. female	1.041 (0.985-1.100)	0.154	–	–	1.094 (1.009-1.187)	0.030	–	–
**Race**								
White	Reference	0.549	Reference	0.016	Reference	0.000	Reference	0.0003
Black	1.048 (0.948-1.160)	0.359	1.268 (1.073-1.498)	0.005	1.273 (1.109-1.461)	0.001	1.539 (1.188-1.996)	0.001
Other	1.036 (0.933-1.151)	0.504	0.959 (0.816-1.126)	0.608	0.877 (0.757-1.015)	0.079	0.915 (0.712-1.175)	0.486
**Marital status**								
Yes vs others	1.038 (0.983-1.095)	0.180	0.983 (0.900-1.073)	0.695	1.156 (1.070-1.249)	0.000	0.973 (0.842-1.124)	0.709
**Laterality**								
Right side	Reference	0.000	Reference	0.433	Reference	0.0001	Reference	0.554
Left side	0.990 (0.938-1.046)	0.726	1.012 (0.937-1.093)	0.761	1.007 (0.930-1.090)	0.868	1.019 (0.888-1.171)	0.785
Both side	1.362 (1.201-1.545)	0.000	0.892 (0.737-1.080)	0.243	2.391 (2.021-2.829)	0.000	1.723 (0.635-4.676)	0.285
**Surgery**								
Performed vs. no	1.898 (1.797-2.005)	0.000	1.920 (1.753-2.102)	0.000	3.778 (3.477-4.106)	0.000	2.630 (2.095-3.302)	0.000
**Radiotherapy**								
Yes vs. no	0.822 (0.771-0.875)	0.000	1.032 (0.924-1.152)	0.575	0.522 (0.477-0.571)	0.000	0.833 (0.641-1.082)	0.170
**Chemotherapy**								
Yes vs. no	1.090 (1.031-1.152)	0.002	1.481 (1.362-1.610)	0.000	0.672 (0.620-0.729)	0.000	0.973 (0.801-1.182)	0.784
**Metastasis**								
Bone mets Yes vs. no	0.709 (0.655-0.767)	0.000	0.830 (0.753-0.916)	0.000	0.402 (0.371-0.435)	0.000	0.590 (0.466-0.748)	0.000

Brain mets Yes vs. no	0.653 (0.571-0.746)	0.000	0.712 (0.612-0.829)	0.000	0.406 (0.357-0.461)	0.000	0.741 (0.526-1.045)	0.087
Liver mets Yes vs. no	0.626 (0.565-0.693)	0.000	0.741 (0.666-0.825)	0.000	0.408 (0.368-0.453)	0.000	0.612 (0.473-0.793)	0.000
Lung mets Yes vs. no	0.760 (0.706-0.819)	0.000	0.865 (0.796-0.941)	0.001	0.399 (0.369-0.431)	0.000	0.543 (0.455-0.649)	0.000
**Grade**								
well, moderate vs poor, undifferentiated	1.074 (0.985-1.171)	0.106	–	–	1.835 (1.561-2.158)	0.000	1.779 (1.499-2.111)	0.000
**Income**								
<60,000 vs >60,000	1.025 (0.961-1.093)	0.459	–	–	0.829 (0.751-0.916)	0.000	0.962 (0.804-1.152)	0.676
**Tumor size**								
< 4 cm	Reference	0.097	–	–	Reference	0.001	Reference	0.004
4-7 cm	0.899 (0.806-1.002)	0.054	–	–	0.996 (0.851-1.165)	0.957	1.350 (0.960-1.898)	0.085
> 7 cm	0.950 (0.860-1.049)	0.311	–	–	1.483 (1.285-1.711)	0.000	1.849 (1.336-2.559)	0.000

Abbreviations: HR: Hazard Ratio; CI: Confidence Interval; ICIs: Immune Checkpoint Inhibitors

Table 3: **PRISMA checklist 2009 for meta-analysis for ccRCC RCTs**

Section/topic	#	Checklist item	Reported on page #
**TITLE**			
Title	1	Identify the report as a systematic review, meta-analysis, or both.	1
**ABSTRACT**			
Structured summary	2	Provide a structured summary including, as applicable: background; objectives; data sources; study eligibility criteria, participants, and interventions; study appraisal and synthesis methods; results; limitations; conclusions and implications of key findings; systematic review registration number.	1
**INTRODUCTION**			
Rationale	3	Describe the rationale for the review in the context of what is already known.	1,2
Objectives	4	Provide an explicit statement of questions being addressed with reference to participants, interventions, comparisons, outcomes, and study design (PICOS).	1,2
**METHODS**			
Protocol and registration	5	Indicate if a review protocol exists, if and where it can be accessed (e.g., Web address), and, if available, provide registration information including registration number.	9
Eligibility criteria	6	Specify study characteristics (e.g., PICOS, length of follow-up) and report characteristics (e.g., years considered, language, publication status) used as criteria for eligibility, giving rationale.	11
Information sources	7	Describe all information sources (e.g., databases with dates of coverage, contact with study authors to identify additional studies) in the search and date last searched.	10
Search	8	Present full electronic search strategy for at least one database, including any limits used, such that it could be repeated.	10

Continued on next page

Table 3 – continued from previous page

Section/topic	#	Checklist item	Reported on page #
Study selection	9	State the process for selecting studies (i.e., screening, eligibility, included in systematic review, and, if applicable, included in the meta-analysis).	10
Data collection process	10	Describe method of data extraction from reports (e.g., piloted forms, independently, in duplicate) and any processes for obtaining and confirming data from investigators.	10
Data items	11	List and define all variables for which data were sought (e.g., PICOS, funding sources) and any assumptions and simplifications made.	N/A
Risk of bias in individual studies	12	Describe methods used for assessing risk of bias of individual studies (including specification of whether this was done at the study or outcome level), and how this information is to be used in any data synthesis.	11
Summary measures	13	State the principal summary measures (e.g., risk ratio, difference in means).	N/A
Synthesis of results	14	Describe the methods of handling data and combining results of studies, if done, including measures of consistency (e.g., I^2) for each meta-analysis.	N/A
Risk of bias across studies	15	Specify any assessment of risk of bias that may affect the cumulative evidence (e.g., publication bias, selective reporting within studies).	N/A
Additional analyses	16	Describe methods of additional analyses (e.g., sensitivity or subgroup analyses, meta-regression), if done, indicating which were pre-specified.	N/A
**RESULTS**			
Study selection	17	Give numbers of studies screened, assessed for eligibility, and included in the review, with reasons for exclusions at each stage, ideally with a flow diagram.	9, Fig. 8. 20
Study characteristics	18	For each study, present characteristics for which data were extracted (e.g., study size, PICOS, follow-up period) and provide the citations.	Table 3
Risk of bias within studies	19	Present data on risk of bias of each study and, if available, any outcome level assessment (see item 12).	N/A
Results of individual studies	20	For all outcomes considered (benefits or harms), present, for each study: (a) simple summary data for each intervention group (b) effect estimates and confidence intervals, ideally with a forest plot.	Tables 3, figure 10, S5, S6 figures
Synthesis of results	21	Present results of each meta-analysis done, including confidence intervals and measures of consistency.	N/A
Risk of bias across studies	22	Present results of any assessment of risk of bias across studies (see Item 15).	Figure 9,11

Continued on next page

Table 3 – continued from previous page

**Section/topic**	**#**	**Checklist item**	**Reported on page #**
Additional analysis	23	Give results of additional analyses, if done (e.g., sensitivity or subgroup analyses, meta-regression [see Item 16]).	N/A
**DISCUSSION**			
Summary of evidence	24	Summarize the main findings including the strength of evidence for each main outcome; consider their relevance to key groups (e.g., healthcare providers, users, and policy makers).	18-21, 24, figure 10, S5, S6 figures
Limitations	25	Discuss limitations at study and outcome level (e.g., risk of bias), and at review-level (e.g., incomplete retrieval of identified research, reporting bias).	8
Conclusions	26	Provide a general interpretation of the results in the context of other evidence, and implications for future research.	8
**FUNDING**			
Funding	27	Describe sources of funding for the systematic review and other support (e.g., supply of data); role of funders for the systematic review.	N/A
